# Supplementary material for: Health worker acceptability of an HIV testing mobile health application within a rural Zambian HIV treatment programme
Source: PLoS One. 2025 Jun 5;20(6):e0312646. doi: 10.1371/journal.pone.0312646 (PMC12140264; doi:10.1371/journal.pone.0312646)
Supplement: S1 File — (DOCX) [file pone.0312646.s001.docx]

# S1 Lynx acceptability survey

Facility: _____________

Please select one answer for each question. For questions with the 1-5 scale select based on if you agree or disagree with the statement, 1 being strongly disagree up to 5 bring strongly agree.

Gender:

Man Woman Other

Age Group:

18-25 26-35 36-45 46-55 56+

**Perceived Ease of Use**

1. Lynx is easy to use

1 2 3 4 5

1. It was easy for me to learn to use Lynx

1 2 3 4 5

1. The application layout is consistent when moving between questions

1 2 3 4 5

1. Whenever I made a mistake using Lynx, I could correct the mistake easily and quickly
   - - - 1. 2 3 4 5

**Perceived Usefulness**

5. Lynx is useful for my HIV testing practice

1. 2 3 4 5

6. Lynx helped me manage my patient’s health effectively

1. 2 3 4 5

7. Lynx improved my access to delivering health care services

1. 2 3 4 5

8. Lynx has provided an acceptable way to deliver healthcare services

1 2 3 4 5

**Perceived Compatibility**

9. I could use Lynx even when the internet connection was poor or not available

1. 2 3 4 5

10. I think that using Lynx fits well with the way I like to work

1 2 3 4 5

11. I have the resources necessary to use Lynx

1 2 3 4 5

12. I have the knowledge necessary to use Lynx

1 2 3 4 5

13. Lynx is not compatible with the way I work

1 2 3 4 5

14. A specific person (or group) should be available for assistance with difficulties concerning Lynx

1 2 3 4 5

15. If I had the opportunity, I prefer working on paper

1 2 3 4 5

*This is the end of the survey, thank you for your participation.*
