# Supplementary material for: Health worker acceptability of an HIV testing mobile health application within a rural Zambian HIV treatment programme
Source: PLoS One. 2025 Jun 5;20(6):e0312646. doi: 10.1371/journal.pone.0312646 (PMC12140264; doi:10.1371/journal.pone.0312646)
Supplement: S2 File — (DOCX) [file pone.0312646.s002.docx]

# Key person interview guide

*Note to researcher: questions are not to be completed in particular order but can be completed based on the natural progression of the dialogue*

Usefulness and Usability of Lynx application

1. What is your experience of using the Lynx system?
   1. Probe on: outputs of Lynx (testing services, testing strategies)
   2. Probe on: why positive or negative
2. How does the Lynx mobile capturing process differ from the traditional paper-based capturing?
   1. Probe on: resources required and available for each
   2. Probe on: time of capturing process, effect of time use (more or less)
   3. Probe on: benefits or weaknesses of Lynx instead of paper
3. How difficult is it to complete Lynx data capturing?
   1. Probe on: time required
   2. Probe on: resources (network, power)
   3. Probe on: software difficulty (education and training)

Compatibility for completing client capturing on Lynx

1. Can you discuss the conditions in which it is more easy and more difficult to complete Lynx capturing?
   1. Probe on: different work settings (rural or urban, facility or community)
   2. Probe on: staff objectives and support
2. What could be improved to increase compatibility?
   1. Probe on: software and hardware of application
   2. Probe on: work setting
3. Do you have any final comments on your experience using Lynx?
