# Supplementary material for: Health worker acceptability of an HIV testing mobile health application within a rural Zambian HIV treatment programme
Source: PLoS One. 2025 Jun 5;20(6):e0312646. doi: 10.1371/journal.pone.0312646 (PMC12140264; doi:10.1371/journal.pone.0312646)
Supplement: S4 File — (PDF) [file pone.0312646.s004.pdf]

R14/49 Mr Andres Luis Larson Montaner

HUMAN RESEARCH ETHICS COMMITTEE (MEDICAL)

CLEARANCE CERTIFICATE NO. M220720

**NAME: (Principal Investigator)**	Mr Andres Luis Larson Montaner
**DEPARTMENT:**	School of Public Health
**PROJECT TITLE:**	Health worker acceptability of an HIV testing mobile health application within a rural Zambian HIV treatment program
**DATE CONSIDERED:**	29/07/2022
**DECISION:**	Approved unconditionally
**CONDITIONS:**	
**SUPERVISOR:**	Prof Pedro Pisa and Prof Charles Chasela
**APPROVED BY:**	Dr CB Penny, Chairperson, HREC (Medical)
**DATE OF APPROVAL:**	04/10/2022

This clearance certificate is valid for 5 years from date of approval. Extension may be applied for.

DECLARATION OF INVESTIGATORS

To be completed in duplicate and **ONE COPY** returned to the Research Office Secretary on the Third Floor, Faculty of Health Sciences, Phillip Tobias Building, 29 Princess of Wales Terrace, Parktown, 2193, University of the Witwatersrand. I/we fully understand the conditions under which I am/we are authorized to carry out the above-mentioned research and I/we undertake to ensure compliance with these conditions.Should any departure be contemplated, from the research protocol as approved, I/we undertake to resubmit the application to the Committee. **I agree to submit a yearly progress report.** The date for annual re-certification will be one year after the date of convened meeting where the study was initially reviewed. In this case, the study was initially reviewed in **July** and will therefore be due in the month of **July** each year. Unreported changes to the application may invalidate the clearance given by the HREC (Medical).

Principal Investigator Signature — Date: 04/10/2022

PLEASE QUOTE THE PROTOCOL NUMBER IN ALL ENQUIRIES
