# Supplementary material for: Health worker acceptability of an HIV testing mobile health application within a rural Zambian HIV treatment programme
Source: PLoS One. 2025 Jun 5;20(6):e0312646. doi: 10.1371/journal.pone.0312646 (PMC12140264; doi:10.1371/journal.pone.0312646)
Supplement: S6 File — (PDF) [file pone.0312646.s006.pdf]

To ERES IRB

We are submitting for an amendment approval to the existing protocol "End Term Evaluation of the Extending Quality Improvement Program (EQUIP)," IRB No. 00005948, FWB No. 00011697.

We have added clarity to objective 1 to include impact and contribution of innovative digital platforms on key programmatic indicators, cited in the objective, that were implemented in the EQUIP program. The modified objective now reads as "*To determine the effect of EQUIP intervention strategies on improving testing, yield, linkage, retention and viral suppression including innovative digital systems and processes*." With the modification we have submitted both the quantitative and qualitative tools that will be used to respond to the modified task. Other changes to the modification include:

1. Additional outcome listed as "*Process and impact evaluation of innovative digital systems (i.e., Lynx) and platforms to improve various program and clinical outcomes*"
2. Addition of the following persons as Primary Investigators: Dr. Ben Chirwa, Dr. Ethan Zulu, Dr. Ian Sanne, Dr. Pedro Pisa, Dr. Eula Mothibi, Dr. Constance Wose Kinge, Andres Montaner.
3. Addition of qualitative Interview and Focus Group Discussion guide on acceptance
4. Addition of quantitative Survey on acceptance
5. Extended timeline to meet the objectives

In our submission we have included two copies of the protocol (one copy includes underlines of all the changes to the protocol, the other copy is a clean version), the additional data collection tools, and the consent forms for the additional tools.

We hope this modification meets your considerable approval.

We wish to thank you for your support and look forward to our continued partnership.

Yours Sincerely,

Dr. Ben Chirwa
Country Director

DG Office Park,13C Bishops Road, Kabulonga, Lusaka
P O Box 30030, Lusaka, Zambia

LCO NO: 120160003000

Directors: Ian Matthias Sanne | Mukwandi Chibesakunda | Peter Mwaba | Kawaye Kamanga
Nonhlanhla Nyewula | Cecilia Bothoboile Mothibi – Wagbafor | Ben Chirwa
