# Supplementary material for: Health worker acceptability of an HIV testing mobile health application within a rural Zambian HIV treatment programme
Source: PLoS One. 2025 Jun 5;20(6):e0312646. doi: 10.1371/journal.pone.0312646 (PMC12140264; doi:10.1371/journal.pone.0312646)
Supplement: S10 File — (ZIP) [file pone.0312646.s010.zip › Transcript_1_deidentified.docx]

**Participant A:** ****Bemba**** Yes he was confirming when he is not clear about a question, so I said I will be translating to him

**Reseacher** : That’s good

**Participant A:** Sure

**Researchers**: Can I first ask how long have you been working in any hospital

**Participant A:** ****Bemba****

**Participant B:** *From 2005 ***Bemba****

**Participant A**: So he started working as a counsellor in XXX and joined right to care in XXX

**Researcher:** So you have lots of experience

**Participant B:** Yes

**Researcher:** And can you start to tell me then what it was like when you started using Lynx when you were counselling what it was like just what you thought of in the beginning?

**Participant A:** ****Bemba****

**Participant B:** ****Bemba****

**Participant A:** So he is saying at first it was a bit difficult to use Lynx, because it was too long and the questions were too many and then looking at the set up of the facility if you have to look at the number of clients the queues waiting you would have to spend more time with one client, but now that the questions have been simplified and some parts have been updated like to loot numbers you don’t have to enter the loot number, only the expiring date that has made the usage is a little bit familiar and user friendly

**Researcher :** Ok and can you tell me more about how using the tablet has maybe affected the way that you would work during the day

**Participant A:** ****Bemba****

**Participant B:** ****Bemba****

**Participant A:** So he is saying previously there were 3 of them using the application but now the other two are gone so its make him work more, he has more work load he has to capture the clients at the same times by himself so it’s a bit difficult for him

**Researcher:** Can I ask his other colleagues also have it so the other counsellors were also able to use it, and so you were not the only one? Can you tell us more about how it was like to work when others also had it

**Participant A**: ****Bemba****

**Participant B**: ****Bemba****

**Participant A**: So it was a bit easy, I was much easier cause if the other counsellor takes a while doing it on their own and then it also became a challenge when the other one started leaving, the tablet had stop working so again it had to come back to him to do the work

**Researcher**: Ok, can you tell me the main difference that you noticed when you were counselling a client just on paper or using a paper register versus when you were counselling a client using a tablet

**Participant B:** ****Bemba****

**Researcher**: Any difference good or bad

**Participant A:** ****Bemba****

**Participant B:** So when using a hard copy it was very fast ****Bemba****

**Participant A:** So he is saying it was easy using the hard copy because it was faster as you are counselling you are able to take the questions when you are done screening you then proceed with the testing, but with the Lynx you have to do it step by step as you are talking you also enter on the tablet if say you wait until you finished counselling the client layer you come back to enter the details because of network it will just be synchronizing telling you to proceed to the next page.

**Researcher**: Ok, so you have already told me a little bit but can you tell me some more about the challenges that you noticed while you were using the tables, so I hear that originally it was a bit too long and you were faced with some network challenges and also some times your colleagues or people from the hospital will send the tablet to get fixed but initially it takes time for the tablet to get fixed, are there any other challenges that you have noticed that have stopped you from maybe using the Lynx

**Participant B**: Challenge number one was the network ****Bemba****

**Participant A:** ****Bemba****

**Participant A**: So apart from the earlier challenges of network he is also saying sometimes the life span of the batteries low because you will find that while capturing the client and the battery is low and if you plug it in the charger the tablet would freeze meaning you cannot proceed with the capturing of the details of the client. So the life span of the battery was a challenge

**Researcher**: Ok, and how about using the tablet it self? If you have a full battery how did you find using the application to just actually typing in like your client details and answering questions? Can you tell me more about just filling in the tablet details outside of the battery or the network, the questions them self.

**Participant A**: ****Bemba****

**Participant B**: ****Bemba****

**Participant A**: Apart from these challenges it works perfectly for him

**Researcher**: OK, Can you tell me more of the differences you experienced when you were using it in the facility versus in the community

**Participant A**: ****Bemba****

**Participant B**: ****Bemba****

**Participant A**: So he says looking at the nature of the tablet it’s big, going to the community you are talking to a client you are busy capturing the client feels uncomfortable maybe you are boastful, you want to show off to them that you have a big phone so it’s much easier use at the facility because they know that this is a facility set up the information that they are capturing will be used at the facility. But when you are in the community it feels like they start questioning why are you taking this information are trying to ****inaudible****

**Researcher**: Ok, can I also aske especially at the facility can you tell me more of the difference for using a tablet on a quiet day versus a busy day

**Participant A**: ****Bemba****

**Participant B**: ****Bemba****

**Participant A:** So when it’s a busy day he doesn’t enter directly on the Lynx application so that it quickens the process he would just use the hard copy to screen into and capture all the details that he needs to use. When the clients have gone he uses that information to enter now on the Lynx application. But when it’s a quiet day he is able to capture directly on the Lynx application

**Researcher**: Ok, and sometimes we can also see on the registers that there maybe like 20 tests coming in for a week and some weeks we can see Lynx also submitted just as many tests as the registers so also 20, but then maybe the next week Lynx has maybe 12 or 25 and then the following week it’s the same again and the week after Lynx is missing some tests that are captured on the registers. So maybe can you think of some examples as to why sometimes you can capture all of the tests on Lynx and other weeks you are unable to capture all of the tests on Lynx

**Participant A***: ***Bemba****

**Participant B**: ****Bemba****

**Participant A**: So like he said he is just alone using the tablet so mostly he only captures the ones he has tested himself and then you find that the other 2 or 3 have gone to the community and have done the test, he is working at the facility he will capture the ones he has worked on. Meaning those ones will be left out and then there are some days that he would just dedicate his time to entering or recording all the clients that were tested by the other colleagues on the Lynx tablet. So you will find some days it will record number of those who are in the register because he would be able to capture everyone and then there are some days when he has just captured what he has worked on the rest would be left out

**Researcher**: Ok, make sense we are almost done, can you think of a way that the Lynx tablet can be improved to better fit the work environment besides what you have Already mentioned the network, the battery, the size and fixing the tablet. Can you think of anything else that could make it easier for you.

**Participant A:** ****Bemba****

**Participant B**: ****Bemba****

**Participant C:** ****Bemba****

**Participant A:** Ok so the first one he said these tablets that they are using to enter the clients information they are also using to make calls to clients so when you look at it they currently receive data bundles they don’t receive talk time where they can convert into minutes and make call the client so he is asking if they can apart from the data they can also be receiving talk time so that they able to communicate with the client and also he is saying that the first part when your are about to register the client there are some repetition there in the questions for an example it will ask you to enter the district you enter the district, then it asks you to enter the hub and you enter the hub so it’s more like repetition and also when you go to the index plan you enter the index name it will also ask you something almost similar to what you have already entered. So if that also can be worked on.

**Researcher**: Ok we can look at it that’s all the questions I have for today unless you have any comments you want to say we can also listen about Lynx

**Participant A**: ****Bemba****

**Participant B**: ****Bemba****

**Participant A**: He is complaining that his friends were complaining that for them they don’t use the tablet for like 6 months it will stop operating the way its supposed to and the 5 months it also stopped but for him although it has continued working it has also given him problems so what ever you bring in the future it should be something that works for longer period of time

**Researcher**: Ok it’s all noted, thank you.
