# Supplementary material for: Health worker acceptability of an HIV testing mobile health application within a rural Zambian HIV treatment programme
Source: PLoS One. 2025 Jun 5;20(6):e0312646. doi: 10.1371/journal.pone.0312646 (PMC12140264; doi:10.1371/journal.pone.0312646)
Supplement: S10 File — (ZIP) [file pone.0312646.s010.zip › Transcript_2_deidentified.docx]

**Researcher**: And just to start this off can you first just tell me how long you have been working in hospitals? Maybe we will start this side

**Participant A**: I have been working here since XXX so that’s XX years

**Researcher**: Always in XX?

**Participant A:** XX, yes

**Researcher**: Ok, and for you?

**Participant B**: XX years

**Researcher**: OK

**Participant B**: I started in XXX, it’s how many years?

**Participant A**: X…

**Participant B:** X

**Participant C:** Since XXX

**Researcher**: So you just….experience between everyone, and how was your experience in working with Lynx as a counsellor? I will start this side this time,

**Participant C**: I know…

**Researcher**: Ok, you can start

**Participant A**: Working with Lynx has been nice, accept, the difficulty I think we encounter I think it’s upgrading of applications sometimes its not done in time, so you would find that the tablet has not been used for some time because of that problem

**Researcher**: And when you mean upgrading, you mean?

**Participant A**: The applications on the tablets, sometimes we need upgrading when you take these tablets to the office they are not done in time

**Researcher**: Oh ok, and how about for you

**Participant B:** In the tablets there is on component which I am not sure, we are component of children, that of children

**Researcher**: Can you ask the question again sorry I didn’t understand

**Participant B:** In that tablet there was only component for adults, but for children it’s better you include also for children

**Researcher**: Specifically for children

**Participant B**: Yes for example Peads, there are no component for Peads, so in years we just go where the adults we indicate age for children

**Researcher**: Ok

**Participant A**: and I think the to add on Peads I think the problem is rounding up years because sometimes you find you have to round off to the nearest one year when a child is six months there should be more closer. That rounding up you find that you have to make people…so it gives an impression that this person is older that here…

**Researcher**: If one of you needs to go you can go I don’t want to keep you

**Participant B**: She is opening a file for a patient

**Researcher**: Ok you can go I don’t want to disturb your work. Ok so I understand that the Peads it could be more specific?

**Participant B:** Yes

**Researcher**: Especially for younger age, and for you how has it been like working with the Lynx tablet?

**Participant C**: Working with the Lynx tablet is ok, at least there is no problems

**Researcher**: Ok, has it affected the way that you work either in a good way or in a bad way…in a good way? How can you explain?

**Participant C**: In a good way its like, its good that every thing we are working on we report

**Researcher**: Ok

**Participant A:** And also it has simplified matters because if we are using hard copies to send this information it would take a long time to reach you, and maybe in the process we loose the documents but this is instant, so it’s good

**Participant B**: Its good

**Researcher**: Ok, and what are the biggest differences between when n you capture on paper versus capturing on Lynx

**Participant B:** Sometime you can lose the paper but with the Lynx you can not lose just like that, but the papers I can misplace it and it be difficult for me to report

**Participant A:** And the paper will pass through many people, our supervisor, the district in the process it may get lose and things like that so redo this, but this is just direct I think it’s good

**Researcher**: And how about the actual capturing its self like When you are writing in the register versus typing in the tablet, is one easier than the other?

**Participant B**: Writing in my note book is easier than typing, because typing I will start with the name and what but in my book it doesn’t even take time to report

**Researcher**: And for you?

**Participant C:** Its just the same

**Researcher**: Do you have the same experiences as your colleagues?

**Participant A:** I think that it has been over come, in that first we are told that as you test you enter but that was cumbersome so when you are done the testing then you do the sending its easier, ok so after you have done the paper work you have done the tests and that’s when you send after, after you are done and all that it’s easier that way, than you are in the ward then you are with a tablet

**Researcher**: Then have you noticed if it’s easier or harder when you are in the community versus when you are at the hospital its self?

**Participant A**: In fact even in the community what we do is get the names here in a book then send them from here at the end of the day

**Researcher**: Ok, so you would capture, even when you are in the community you would capture from here or once you get back here

**Participant A**: No what I mean is you would get the details of that person the clients and then when you come here that’s when you enter all that information now for us to send

**Researcher**: Ok, so is there any difference for you using the tablet in the hospital versus in the community?

**Participant A**: The difference in the community first of all people get suspicious you are talking to them you are using a tablet, they ask why are you using this where are you taking this information so this is why it is much safer you just capture in your book and then you come and enter in the facility

**Participant C:** Sometime you can have 5 clients you never know with the tablet it takes a bit of time you cannot finish those people at that time, so when I write on my note book I come back sit down and start entering and it’s ok

**Researcher**: Ok, I am just asking the difference between using the tablet in the hospital versus in the community for you is it any different?

**Participant C:** Its ok if it’s not broken

**Researcher**: But when you use it would you use it differently if you are testing someone in the hospital versus testing someone in the community

**Participant B:** ***Bemba***

**Participant C:** ***Bemba***

**Participant A**: Same scenario, first in the note book then they come and enter here

**Researcher**: OK, and you? I will ask each one

**Participant B**: Its just one and the same, it’s not good for our clients when we are doing counseling I am asking questions and I am busy on my phone writing, so that based on our client I think they would not be comfortable, they would be suspicious

**Participant A**: They think you don’t care you are on your phone

**Participant B**: ***Bemba***

**Researcher**: That is not nice. So I hear about this challenge you have of people being suspicious, is there any other…either with your clients or with your work or even the tablet it’s self something that makes it difficult to complete Lynx?

**Participant B:** No

**Researcher**: No? You can think of any challenge

**Participant A:** Ok the challenge I have experienced is on the location or the addresses, you know we don’t have in our setting the house numbers, streets so that becomes a difficult challenge, we just have maybe the residential but we have no street name no address so that is a challenge there

**Researcher**: Ok, and for you?

**Participant B**: It’s the same

**Researcher**: anything else

**Participant C:** No I don’t have a challenge

**Participant A:** Maybe the other challenge is that I would want the tablet is more inclusive in service because now we are just using it as a tool of reporting, but then we have other tasks we do like in retention of our clients and we need to call them, but then the tablet is just the data we don’t have anything for voice calls so that we arrange with this person where can we find you, where do you reside and where can we meet. And as I said we went to community there are no streets and you have to keep calling where are you I am here, so this has no provision for talk time. It’s only data bundles so which make the other component of retention of clients difficult because we have no means of. So if maybe also talk time would be supplied into the tablet so that we can also use it for the component of retention

**Researcher**: Ok, I mean I am not in charge of that but I will include it in the reporting, and so as soon as the Lynx tests come in they can be viewed instantaneously, as you were saying, we can see that in some months almost all of the tests on the registers are also on Lynx but then in other months there are some test that are missing from the Lynx side but we se them on the register but then maybe even the next month the numbers are matching again so can you think of a reason why sometimes all the test are coming on Lynx

**Participant C:** ***Bemba***

**Participant B**: Sometimes the Lynx component doesn’t work you find that you go to the offices to report that my Lynx is not working but when you get the tablet sometimes it takes longer, for an example me I don’t have a tablet my charging system is not working, so my tablet has been there for closed to 2 months I have not received my tablet, so for me it’s difficult to report

**Researcher**: Sure

**Participant B**: Yes

**Researcher**: So like if there is an issue with Lynx it would need to be fixed quickly

**Participant B**: Yes otherwise I have been meaning to report on Lynx, so I am happy to see you have come maybe you are the right person to encourage them to give me back, or to give me there are some tablets maybe there they can help me to give me one so that I can also do the reporting because I am still in the system

**Researcher**: Ok, I mean I dont work in Zambia anymore I am kind of outside at this point but…

**Participant B**: Even if you are outside you can do something

**Researcher**: I can give them the message

**Participant B:** Yes just the message

**Researcher**: And from others? Again I am just asking how some months we can see how all of the test from the registers are also on Lynx but then the other months maybe not all of the tests from the registers are on Lynx, so is it a technical issues maybe they are not getting drop back to you maybe they are not fixed quickly enough?

**Participant B:** ***Bemba***

**Participant A**: Because there was one time you would be sending but then you are told you are not sending, so you don’t know what happens there, because you thought you are sending but the report would say you have not been sending, you see

(inaudible)

**Participant A**: We have instances that you are told you have not been sending anything, but I have been sending

**Participant B**: Me when I have been sending with the tablet, maybe I have sent 5 but when it come down on record it tells me that I have sent 2, so there we don’t know what happened

**Researcher**: Ok, that’s interesting…

**Participant B**: But we try by all means to send the to use Lynx, we try by all means

**Researcher**: And then is there anything no changes from your side, but is there something you can recommend to me, or to like Right to Care in terms of your work responsibilities or the tablet or the way that you are working that would make it easier for you to have the time or just to be able to complete Lynx

**Participant C**: Yes its easier

**Researcher**: Can you explain

**Participant B:** Its easier because immediately when I am reporting I am using the Lynx, immediately when I enter someone’s name it has already reporting to that place that is the most important thing, because the report reach you very fast

**Researcher**: So is there anything that we could do to make it easier for you to report

**Participant A**: I think one thing I think you have already did at, one time we were reporting birth dates for people but that is a bit long but when you included the option of yes you have made it simpler, because it would be 39 date month so they add a simple option

**Researcher**: Can you think of anything else either from the tablet or again from the way that you have your work experience

**Participant A:** Ok like she said I think let’s close it down on Peads, let be slightly exactly rather than rounding up a person who is 5 months or 6 months to one year, that difference is too much that gap is too much

**Researcher**: OK

**Participant B**: This is too much

**Researcher**: Ok, anything from you? You have been very quiet. If you could tell the bosses some where to make Lynx better what would you tell them, or the tablets anything like that

**Participant C:** For me I think ( inaudible) because there was a time I find that when***Bemba*** when you are testing it would count minutes from 20 ( inaudible) but for now at least you have made it easier (inaudible) it was taking time so you would find that I still have 10 clients to enter on Lynx, you find that…

**Researcher**: Ok so we can make sure that we keep on checking with our counsellors so that if anything changes the testing practices like the new age groups removing the timer and things like that to make the Lynx easier and easier to use hopefully

**Participant A**: And I don’t know whether there can be a provision where we can send some recommendation if we think maybe we could change something, just suggestions because there isn’t that provision

**Researcher**: Ok that makes sense because when you send it to like the HTS coordinator or something and then if they agree then we can approve it and we can do it, we should be able to do that

(Inaudible)

**Participant A**: But when we need to maybe to change some things to make it more friendly more user friendly if we have recommendations there is no provision on that one we have to talk to them verbally and that sometimes is not attended to

**Researcher**: That is a good one

**Participant A**: Thank you

**Researcher**: That is all the questions that I wanted to go through and do you have any final comments? For improvement challenges anything else

**Participant A**: I think in my side I have covered what ever I had to say

**Researcher**: Ok

**Participant A:** No additions

**Participant B:** No subtractions

**Participant C**: I have no addictions

**Researcher**: Ok,

**Participant A:** Other wise it was a good innovation when you came up with the Lynx program

**Researcher**: Ok it’s good to hear

**Participant A**: It was a good innovation to man kind

**Researcher**: Ok
