# Supplementary material for: Health worker acceptability of an HIV testing mobile health application within a rural Zambian HIV treatment programme
Source: PLoS One. 2025 Jun 5;20(6):e0312646. doi: 10.1371/journal.pone.0312646 (PMC12140264; doi:10.1371/journal.pone.0312646)
Supplement: S10 File — (ZIP) [file pone.0312646.s010.zip › Transcript_3_deidentified.docx]

**Researcher:** Ok so as we start can you just tell me how long have you worked as lay counsellor? To start this off we can start on this side

**Participant A:** Since XXX

**Researcher:** Ok and for you?

**Participant B:** The same almost, more than XX years

**Researcher:** Ok

**Participant D**: XX years

**Researcher:** XX years

**Participant E:** XX years

**Researcher:** XX years? So we have XX counsellors with us. So can you tell me, I will start this side, can you tell me how your experience are using the Lynx as a lay counsellor?

**Participant E**: It’s a good device it helps in working very good also its makes it easier to pass over information to relevant people using Lynx

**Researcher:** Ok, and has it affected the way that you do your counseling?

**Participant E**: No it hasn’t in fact it helps because when you, for instance you test at times you are recording using Lynx and also remember and also it helps for other people especially you know where you are they can easily identify you

**Researcher**: Ok and how about for you?

**Participant D:** For me it’s about the same but sometimes when we start to use that Lynx it was difficult to do it but now I understand, so in my experience I see that when I test someone (inaudible)

**Researcher**: So in the beginning it was a bit challenging but you were able to work it?

**Participant D**: Yes

**Researcher**: And how about for you?

**Participant C:** For me its fine, yes but as she had said the first time we had difficulties but currently it’s fine

**Researcher**: Ok…

**Participant B:** Like they said it really has been nice especially when we are keeping records even sometime when we go in the field when we are carrying a tablet it instead of carrying books you just carry the tablet, and in keeping information it really helped very much

**Researcher**: Ok it’s good to hear

**Participant A:** For me at first it was difficult to have even tablet but for now we only do it, and it is very good to have the tablet especially in the field, at the facility we don’t we only do it after our work we enter in our tablets

**Researcher**: Ok, can you tell me more about the difference using the tablet in facility versus using it in the community?

**Participant B**: In the community when you follow clients maybe it’s one or two, it is easier to do testing and enter in the tablet and in the facility you have a lot of clients so it is not easy to use the tablet the same time

**Researcher**: Ok, can you explain more why its not so easy to use it while in the facility

**Participant B**: Time

**Researcher**: It’s the time?

**Participant B:** Yes

**Researcher**: ( inaudible)

**Participant C**: Maybe also at the facility you also have to enter in the register the client who queue there at the facility, then after entering the registers that is when you come and enter in the Lynx

**Participant B:** So then we transfer that information to the tablets the Lynx

**Researcher**: Ok, that makes sense. And then for you is it the same as with your colleagues?

**Participant D:** It’s the same

**Researcher**: It’s the same?

**Participant D:** Yes

**Researcher**: And do you ever find it difficult to…do you ever find the time where it is actually difficult to use Lynx you know because something with the tablet or something with work? I will start this side

**Participant E:** As hinted to you before especially with the facility like this there is too much work to do mostly we use registers and unlike in the field, there in the field we use the instrument because the clients that we normally attend to are individuals you take your time and talk to them and discuss with them counsel what ever

**Researcher**: Ok so it’s sounds like it’s more difficult at the facility because, is more busy or is it because you do more than just testing or is it because you test a lot of people

**Participant E:** First the ( inaudible) you can see we attend to those coming for those who are on PreP they come ( inaudible) at the facility

**Researcher**: Ok, do you ever not have time to capture some one on Lynx because of because of work or something else…

**Participant D**: Sometimes it’s because of work and some times it’s clients

**Researcher**: Ok, and for you

**Participant C**: Yes because when you are in the facility it’s difficult to enter in the register and you see the other one, that one so it takes time so after entering in the register that’s when we come now to the Lynx and we enter the information to the Lynx, it’s fine

**Researcher**: Ok,

**Participant A:** I think it’s the same, and some times we do enter when there is no it’s all the same

**Researcher**: Ok, and how do you feel about how much time it takes to complete Lynx, it’s ok I am sure I can still hear you

**Participant B**: It takes a lot of time but we have to follow the instructions

**Researcher**: Do you think in order to make it fast if we potentially take out some questions, so would you rather have us take out questions or would you rather have us leave the questions in to make it easier, which one do you think would be better from your experience? We could start this side

**Participant A**: From my experience I always follow instructions

**Researcher**: Ok, so you wouldn’t think we should remove any of the questions, because we can if you think we should

**Participant D:** No the questions are Ok, I think they are Ok ( inaudible)

**Researcher**: Ok

**Participant B**: Yes questions are Ok

**Researcher**: So you think the questions are still helpful

**Participants**: Yes

**Researcher**: Ok, also for you too?

**Participant E:** Yes because as it is ( inaudible) have been done today some of the questions have been removed they are not necessarily because some are a duplication of information but as it is now its ok

**Researcher**: Ok, sometimes we can see as Lynx is getting submitted, some months test are submitted and some moths not so many are being submitted but then the following month again more people are submitting on Lynx, do you maybe know why some months people are able to submit more on Lynx and some other months people are unable to submit so much ? Let’s start back this side

**Researcher**: I will say the question one more time, because some times we can see how many coming in from XX and we can see even in September there is twenty tests signing in but then in December there is only five but the in January we are seeing fifty, so do you maybe know why sometimes not so many people are able to submit but then other you are able to submit all of the tests

**Participant B**: I think it depends on the people you test, sometimes we test more sometimes we test less, another reason, I can say because of our tablets sometimes the Lynx stops working

**Participant E:** The time you were here checking on the performance of the staff now when you say ( inaudible) at times there are no reports and sometimes there a few reports because as it is now some of the questions they are not relevant they were very irrelevant before

**Researcher**: Ok, so could you still think of that maybe a week, even now with the improvements, if there is a week where you were not able to capture all of your clients on Lynx, is it only because the tablet is not working or is it because your shift was too busy or what kind of challenges that maybe you would face?

**Participant A:** I can’t understand the question can you repeat it?

**Researcher**: I am just again trying to understand more like sometimes maybe on the registers like today you have captured twenty but then on Lynx we see only five for XX, but maybe tomorrow you are able to get 25 and also 25 on Lynx but then the next day there would 25 on the registers and only be 12 on Lynx, so what could be just the reasons that do always come on Lynx because we talked about the time that maybe the tablet is not working so I trying to understand if there is any other challenge

**Participant E**: Maybe on that one because each counselor has… one should work at the hospital the others should go on the field maybe that one who is at the facility as we are sending… maybe because of the nature of work, so maybe he or she will only enter one of two due to time or pressure of work so that maybe the next day that is when you capture because those she hasn’t entered on the tablet, you enter the following day. Is it possible or is to not good that you cut the client today and you enter tomorrow, that is maybe the problem

**Researcher**: Ok that makes sense I mean as long as they are eventually captured its still good its better if you can do it the same day so you can see how many you get on a Wednesday versus on a Thursday. But even if you put Wednesdays clients on a Thursday as long as they are still there to the hospital. So how is…could you think of something to .are it easier to use Lynx, like a way to improve capturing either it doesn’t take as much time or anything to make it a bit better

**Participant E**: I have said earlier on…for instance we have departments we have also there are days that they record more clients other days they probably make (inaudible)

**Participant B**: ( Inaudible)

**Researcher**: And for you sir can you think of anything to improve it or make it easier to use?

**Participant C**: Yes anyway on that one we have to improve yes, we have to make sure we capture and enter all the client we have so that we don’t have many to call because the next morning or the next we have to work on the we test the we counsel them and we capture the same day, we try to work on that one so that we don’t work for the next day

**Researcher**: Ok, so then you outside of this then have you seen any changes from your side, so if you work the same way is there anything we can do either on Lynx or the way that you are working during the day just to make it easier for you to able to capture so you wouldn’t have to work any hard, what can we do to make

**Participant C**: No it’s fine it seems you have reduced some of the questions so for now all is fine the questions have been reduced like the first one it had more questions and it took time for us to enter. Maybe I just one person it took time for me to capture that client, so we thank you for have you reduced for now

**Researcher**: Ok

**Participant B:** The other thing maybe the other challenge maybe hasn’t come out is the issue of reduced the name registration every month so there times maybe the application is not working so it would stay for a long time and maybe they bring the tablet to the office maybe increase the registering because it disturbs them

**Researcher**: And do you need network to reregister?

**Participant** : Because there are times when the application is not working and we are also busy, or maybe they bring g the tablet from the office, I wait on them so for the tablet to reach them back sometimes so maybe if you can extend the period on when we should do that in registration maybe twice a year I think k that gave hope

**Researcher**: Ok, because at one point we extended it from the 30 days to 90 days to give you more time because we do the goal to reregister is to make sure that you still have the same tablet that you were given so that if you somehow swap tablets when you re-register we could see who was using the tablets and so we wanted it so you re register in 3 months just so we can check that the same tablet is still being used by the same counsellor, but we can try to find a way to make it easier

**Participant B:** Yes I think maybe after 6 months so that could be better

**Researcher**: Ok and how about for you is there any way we can improve the system to make it easier for you

**Participant D**: I think for me as well especially when the tablet is ok there is no problem

**Researcher**: For you?

**Participant A**: For me the same ( inaudible)

**Researcher**: Ok, that’s all the questions I have unless you have any final comments to say

**Participant C**: Maybe I will follow what the madam was saying here, would it be possible for you maybe you hint us to reregister at all it closed down then we can do it for ourselves

**Researcher**: We should be able to….I think maybe I will put it on a piece of paper with pictures and steps on how to do it, so that when you send it to XXX or XXX here they can also have the instructions just so that it is easy and everyone knows how to use it

**Participant C**: It would fine

**Participant E:** Just to come back to the issue of giving it to them there is a discretion because the sad part of the password some, they can’t go to play store for their password so that they do not try and misuse them so if that can be possible from their end because of those passwords

**Researcher**: Oh ok maybe we can discuss…

**Participant C**: Or maybe their PC’s we can give our PC so she can do it

**Researcher**: Ok, that make sense, does anyone else have any other last comments, you two anything else, and from this side

**Participant B**: We were just saying thank you for your Lynx the work is at least easy for us
