# Supplementary material for: Health worker acceptability of an HIV testing mobile health application within a rural Zambian HIV treatment programme
Source: PLoS One. 2025 Jun 5;20(6):e0312646. doi: 10.1371/journal.pone.0312646 (PMC12140264; doi:10.1371/journal.pone.0312646)
Supplement: S10 File — (ZIP) [file pone.0312646.s010.zip › Transcript_4_deidentified.docx]

**Researcher**: Ok, but I will go through a set of questions to guide the discussions, yes thank you. So first just as quick ice breaker, can you just tell me how long you have worked in public health

**Participant A:** Can I quickly start I just started at Right-to-care for XX and here in the (inaudible ) hospital and I have experienced more about Lynx and the thing that I was claiming about this Lynx when you went out to the community you normally test people when we find a positive we document in the Lynx, immediately when we document you we get the addresses for the patients to also get some families report in some linkage and then from that you do submit and after submitting it will go through to the office or the main Right-to-care just things like that.

**Researcher**: Ok, and can you tell me how long you have been working at hospitals

**Participant B:** XX

**Researcher**: XX?

**Participant B**: Yes

**Researcher**: And what has your experience been using the Lynx tablet

**Participant B**: Same as he says

**Researcher**: The same so far?

**Participant B:** Yes

**Researcher**: Ok and I will ask how has it affected your work?

**Participant A**: Its affected because we are using the code in the month of March, because the code that we are using is the XXX, but then when try to log in it has stopped functioning its reduced to the formal code, it was and now it has affected our job we don’t know how are we going to help people we stopped that so it is the most thing that we are (inaudible) right now

**Researcher**: Oh I see ok, and what about before you were blocked from using the tablet, so when you were still using Lynx, when you were using Lynx how did it affect you work during the day

**Participant A:** For me about that I can say it was functioning good without any thing that we can call consequences to our department, so Lynx is good as well as it

**Researcher**: Ok, and how about you before you we locked out of your tablet when you can still sign in how was it affecting your work, did it affect the way you were working?

**Participant B**: Yes

**Researcher**: How so?

**Participant B**: The same as he was saying

**Researcher**: The same?

**Participant B**: Yes

**Researcher**: I think I am going to have to ask you first next time. So how does capturing clients on Lynx, how does it differ from when you were capturing clients on paper? Maybe this time I will ask you first, this side

**Participant B:** I beg your pardon?

**Researcher**: Just the difference between capturing a client on Lynx on the tablet and when you were capturing a client on paper

**Participant B:** The paper is easy uh what can I say to send the messages to the office but the writing is very difficult

**Researcher**: Why, just because of…..

**Participant B:** Writing has many (inaudible)

**Researcher**: The offices?

**Participant B**: The best one is the Lynx

**Researcher**: Ok, So you first have to write it then physically give it to someone and as for Lynx you just put it on Lynx?

**Participant B**: Yes

**Researcher**: Ok that makes sense. How about for you?

**Participant A:** For me? I just go through the tablet because writing would be difficult if you are me I can see because if go in the field or in the community I just tested people, I find today 4 positive and 3 positive instead of capturing in the register but I go through it past the linkage because when I go through the linkage I go to the use after that I open I go in submit to the main office so that immediately when they open they see that the hospital was working as a counsellor, so I don’t even like they are just using the register and the Lynx has stopped working right now so that I can make a request to the office in the login system so that Lynx would start working again, because I have seen that is the biggest challenge I have because the time that I was using Lynx it was performing but as of now even we had offices from Lusaka, I don’t know because there are some places ( inaudible) many are just using the Lynx they can help us as they are performing this and that, oh today you had 5 positives, today you have 3 index today you have 5 ( inaudible) So you can have this now for you to come from there me I can restore my information that I was working on the field put in the register with out linkage that showing that it can take time, but once I was just using the Lynx you can be sure that is what I have been observing about this

**Researcher**: Ok makes sense and then let’s see what about any sort of the resources that it would take to use Lynx, are there any of you…in terms of the times that it would take to complete powerup to stay charged, or the network

**Participant A:** Actually we have that challenge of networks, once we don’t have network it given that we don’t have power to cut of power because it’s a load ( inaudible) so network sometimes you can it can go for a day, us we can wait until you do report for the Lynx, we do say no we can’t do report for the Lynx we don’t ever say no we can’t do the report. So if the power is gone today the network is gone today and it will come tomorrow we make sure that immediately in the morning we start to enter all the in the previous day so that the document it goes in the Lynx so that you submit it

**Researcher**: Ok so if there is a power problem you put it in the day and once the power is back?

**Participant A**: Yes

**Researcher**: Ok that makes sense, And do you have any challenges? So assuming that you have power and you are able to use Lynx normally, what challenges do you face if you had to go to the community or if you have to here at the facility that would make it harder for you to use Lynx

**Participant A**: There is nothing that I can say, some clients are difficult in terms of understanding. Once I test them then they say I think this person comes to steal something about us and even they ask you why are you here you came to give us some money? Because you just came to get our bloods and for us being a counselor we have got confidentiality we have got that power to challenge the client to make him understanding what we are about or where we are going to help them, so now I tell them we are here to test you. Once we test you we have to take information so that if anything happen me I am not around maybe someone can be able to come and identify were you are your place because this village you are going to give me if you are staying in Stambo, you give me Stambo so that someone can come back and see you again even if u am not around. We are not here to kill you, we are not here to steal your property so these things most of the time is happening offer some challenges because being a good professional and being a man you make sure that you are good to them and you give good information and they accept your explanation

**Researcher**: Oh ok that makes sense, have you got anything to say how about you have you faced any issues or challenges when you go through and you are putting a new client on Lynx

**Participant B**: There is big challenges

**Researcher**: Which challenges?

**Participant B:** As (inaudible) not working..

**Researcher**: Ok, but can you think of anything else or is it just the same? Ok it’s fine. I have noticed previously when like earlier last year or this year when Lynx was still working for your facilities sometime I could see that lots of people are using Lynx so I could see the coming test and would get on each day so maybe January it’s very high and then February come and I could see that not so many people and then in March they start to go up again, this is just an example. I have seen that in different months it can you guess or do you maybe know why sometimes it’s use more and some times it’s used not so much?

**Participant A:** Actually I can say that sometimes as we were talking about the challenges so you can see a lot got tested but next month in a few week you can see the other test more because sometimes you can get quiet in terms of network, so today maybe the so whilst you do that today you test but you don’t have network, so sometime so don’t network, so tomorrow when it comes tomorrow then they have just forgotten to come back and to enter in the Lynx so also it can bring us down so also like your performance is not well because over the network issues

**Researcher**: Ok,

**Participant A:** Yes

**Researcher**: So you think it’s mainly network issues

**Participant A:** What I am saying is sometime you can be able to save people and then after that you seems like performance is gone down and different things so us here we have a challenge of networks, so once you see something like that today we are no reporting tomorrow we are not reporting maybe it’s the challenge of networks, like maybe some counsellors they don’t know how to use Lynx, they don’t know how to use it well, so in terms of submit they don’t know so if they fail to capture or submit it can be also I think that

**Researcher**: Ok it makes sense so there is the network there is also the counsellors don’t know how to use tablets

**Participant A**:…they don’t know how to use tablets, yes

**Researcher**: So what about the way thing are in the facility where there is an extra activity or a policy change or something, so something based on the way that the hospital works or the way the community is happening did that ever affect the way that

**Participant A:** It does not affect

**Researcher**: Ok really? Would you use the tablet more or less if it was a busy day especially at the hospital? Because like maybe on a quiet day you have more time but if it’s busy with lots of people would that affect how you would capture on Lynx?

**Participant A:** Actually when it is busy we normally wait like when I come to work I come with my report, sometimes I don’t even enter the community we just come and wait, maybe they come after (inaudible) then I come with my report, so that you submit the report. Like me I know to those who are not even comfortable with Lynx but for me (inaudible) now not even a tablet

**Researcher**: Because the tablet…like it fell?

**Participant A:** It fell yes

**Researcher**: Ok, so that next time someone should get like the protector type of thingy

**Participant A**: Yes, actually they came with that thing it was just like an instant protector so after I just submitted to XXX the one who look up on the phone, so I just call him and they said no we are going to give you back your phone so that you going to have to use Lynx

**Researcher**: Ok that makes sense, that is just about everything I was going to ask. Would you use it… I will ask this one first. Would use Lynx differently in the hospital and in the community? Would you use it differently in the hospital than you would use it in the facility, I mean community sorry

**Participant B:** Yes community, community because in the community its easier

**Researcher**: How come it is easier to use in the community that in the facility

**Participant B**: Because in the community we are free, all that PA says but some we are disturbed by people coming in this room coming in and out walking, but in the village you are free

**Researcher**: Ok that makes sense, and for you

**Participant A**: For me I can to say it both because if I go in the field in the community I go test people and then if I left my phone in the facility I have to come back immediately. If I at least did five, if I have five index, I have to make sure to keep the right paper so that once I reach in the facility I start now open the Lynx myself I still someone even if it’s not busy outside I open the tablet start make sure that I submit everything, just on that when you go in the field you can help maybe like if it is, when it’s raining even if you don’t have an umbrella, so for me it’s a challenge to go with the phone, this is smart phone, this is one of the reasons my phone broke because I just went with the tablet on the field so I was just turn for a minute it fell down, so for me if I come back and leave the phone or I can go with it in the community I do report. And then if it is not easy for me to take care of my table I have to make sure that I capture everything that I have tested or that I have got from the clients I come back to the facility I start to enter

**Researcher**: Ok makes sense. I think we have gone through all of my questions, except maybe you have said that Lynx is usually good and works well, what about it that makes it work well

**Participant A**: Like I said it works well in terms of the main office to know we are working, because it takes time it can be to the counsellors. So we are just using the Lynx maybe it immediate to see our performance. It help us and it helps the main office to know the counsellor is working into the facilities

**Researcher**: Ok perfect it makes sense, and for you?

**Participant B**: Its still the same

**Researcher**: The same?

**Participant B:** Yes

**Researcher**: Ok, and do you have any final comments, any other challenges that you face with Lynx or ways to improve it

**Participant A**: Actually you said the phone change, just don’t understand the question there

**Researcher**: Yes basically that especially a way that it could be improved to fit this hospital better or the community

**Participant A**: The thing about the Lynx is that recently now we don’t have them so we are waiting for them and then it’s still came since it stopped working maybe its been 9 months, so for us it can be a challenge so we have stopped for a long time the report for the Lynx, so this can affect our work to the main office so if the main office can help us by bringing us back in the normal system because our job for the public has stopped, so what is remaining now all we do is clock in and clock out but for that main purpose of that is to report.

**Researcher**: Ok, so how would you…in addition to work faster and to give report to the center office how else would you improve it

**Participant A:** A lot hey that is why I said it help us and the office to see the counsellors how they perform because and it is still difficult to identify the counsellors who are struggling at the office if they can be able to see what you are going through in Lynx it can be helpful to the office that this counsellor is performing this and this today. So once you do this the office also gets affected it can take time to give them the information that is why we said the register because the Lynx is better but you also have to write in the register because some information it can be other people from Lusaka or (inaudible) they come to check they don’t go through the Lynx they just go through the register they start going through and through they check and check

**Researcher**: Ok, and would you want to change anything on the system or the way that the reporting is set up to make it easier?

**Participant A**: No if you say you want to change the system it’s ok, if you say you want to change the system us we have nothing to say we hear from you, you tell us you have to do this and this, maybe you tell us today maybe you come with a good system we make sure that we do everything you tell us

**Researcher**: Ok its good to hear that, what can we change to make this a new better system in the system

**Participant A:** You said what could be..?

**Researcher**:…Changed or improved in the system

**Participant A:** In the system?

**Researcher**: Yes

**Participant A:** Like for now?

**Researcher**: Either in Lynx or the way that the reporting is set up even at the hospital

**Participant A:** Everything is ok its just the challenges

**Researcher**: But what challenges were you faced with?

**Participant A**: Like I said earlier for us we can be that can be able to see how we perform, then it’s easier for us so that we can be able to see it’s easier to use the Lynx and it’s very good

**Researcher**: Ok, and anything else from you?

**Participant B**: No
