# Supplementary material for: Health worker acceptability of an HIV testing mobile health application within a rural Zambian HIV treatment programme
Source: PLoS One. 2025 Jun 5;20(6):e0312646. doi: 10.1371/journal.pone.0312646 (PMC12140264; doi:10.1371/journal.pone.0312646)
Supplement: S10 File — (ZIP) [file pone.0312646.s010.zip › Transcript_5_deidentified.docx]

**Researcher**: Before we start can you tell me how long have you worked at the hospital?

**Participant A:** I work for almost X years

**Researcher**: As a lay counsellor?

**Participant A:** First I work as a volunteer counsellor, now I am a full time lay counsellor, so as a lay counsellor I have worked for one year and some months

**Researcher**: Ok

**Participant B:** Yes I have been here at the hospital for almost X months now, I have worked for Right to care for X months as a professional counsellor, X month lay counsellor and X months professional counsellor

**Researcher**: Ok thank you, and can you tell me about your experiences using Lynx

**Participant A:** Yes Lynx it saved me previously it saved us for reporting especially when we are in the field we report through using Lynx

**Researcher**: And how did it affect the way that you work?

**Participant A:** The only challenge, you find sometimes it’s the network, that is the only time because if we go when I am on the field I go far sometimes the area that I am at has no network it give us a challenge for reporting

**Researcher**: Ok, and for you?

**Participant B:** Yes so for the short period of time that I have used Lynx which is just a month I would say Lynx is just a good gadget to work with, or help to work with. Because it really help us to know where we need to do more of our services, for example we using it to get were we have more positives then we mop out that Hot spots, so it’s really helping us in that way, but the challenge we comes in where we are going for limited places¹ for network challenges because internet service is limited to service down area this small area. But when you go in the out scates there is no Internet so it’s hard for us to go in the field and detect maybe where that location is, so that is a challenge. And another challenge is that our tablets doesn’t really work for…like I would say after they worked on them they worked a little bit of time , even now as we are speaking they are not working the Lynx so that is really a challenge to come work on them, just a month or two the stopped functioning so again that was a bit challenging on our part, but we’ll no it’s a good tool t was effective at time it could give us accurate information that we could get better results

**Researcher**: Ok this makes sense, can you tell me how it’s different to capture a client? For you the way that you are working between capturing it on Lynx and when you come and capture it on the register? How is it different?

**Participant A:** The difference is like when I finished working on Lynx, reporting on Lynx immediately the supervisor will get the report without wasting much time, but when I come and register on the register it takes time to come and capture there after reporting

**Researcher**: How about when you are actually putting the client in whether you are typing or whether you are writing, which one can take more time or which one is easier to record?

**Participant A:** Its on the register because Lynx takes so many questions to complete, yes

**Researcher**: Yes I understand, have you had the similar experience?

**Participant B:** Yes register is very much easier we enter a very few details the Lynx ask you this and that so it takes a bit more time and a place where maybe an internet is a challenge for you to complete just one entry it takes too much of your time as well than the register

**Researcher**: Do you think the extra questions are they useful, or do you think we should remove some?

**Participant A**: No they are useful, all questions are very useful

**Researcher**: Ok, how about for you think we could remove some or…?

**Participant B**: Not really because those questions also serve as… are important purpose for us knowing where we should also look at how look at so we should not remove them just like working on them just so like there might be some kind of have an improvement work on our part. That could help us

**Researcher**: Ok, that makes sense and would you say that it is difficult to use Lynx like the actual like the typing and the button pushing, I think you me mentioned before or you touched on this a little bit, but do you have challenges just when you actually type on Lynx or are you able to do it?

**Participant A:** There is no challenge

**Researcher**: Ok, and for you

**Participant B:** No challenge at all

**Researcher**: Ok. And would you say you use it more in the facility or more in the community? Lets start with you this time

**Participant** **B**: Usually it has been used more in the community, why I say so is more of our work it’s to do with the community so here we receive less number for those that are coming for testing and the like, so usually we find them in the community so that is why we even face those challenges of the network, but if it was at the facility that wouldn’t be the matter at all.

**Researcher**: And can you tell me again why you use it more in the community than in the facility?

**Participant B**: We use it more because that is were we do more of out work

**Researcher**: Oh ok

**Participant B**: Yes mostly counsellor are always in the field

**Researcher**: Ok

**Participant B:** So (inaudible) in a counsellor one particular counsellor and we receive less number of people that are coming for HIV testing and counselling, so most of our testing are done in the community so that is were turn to use it more cause that is were we are doing more of our activities

**Researcher**: Ok that makes sense, and how about for you, do you use it more in the facility or more in the community

**Participant A:** Yes yes

**Researcher**: Is it the same?

**Participant A:** Sure, and after this interview I will go in the field for the same program

**Researcher**: Ok so you mainly work in the field?

**Participant A**: Yes

**Researcher**: And back when Lynx was working we could see that a lot of the times there was more people in the register with the recorded test, that were than in Lynx sometimes we could see lots of people submitted their test on Lynx, but sometimes we could see that not so many people submitted on Lynx but then the following month more people would submit on Lynx do you know why, maybe one month more submissions are coming and then the next month maybe not so much, must I repeat?

**Participant** A: Please repeat

**Researcher**: We have seen, even if there is lots of tests The number of tests that are actually recorded on Lynx compared to the registers differ, on Lynx sometimes there is lots of tests and sometime there is not so many test, even if the hospital registers are getting lots of tests, I hope I am not saying this the funny way…

**Participant A**: The problem is about the network I think

**Researcher**: The network?

**Participant A:** Yes, because as we said previously if we are in areas we’re network is very difficult to get, that is where you would find that sometimes the reporting on Lynx are just low but on register they are high

**Researcher**: Is it? Can you think of anything else beside network, maybe there is nothing else but I just want to ask if there is anything else besides network, like maybe it’s your work responsibilities or it’s too busy or you are focusing on something else

**Participant A:** No the only thing as we said the time I am reporting it takes almost one hour to complete just for one person to complete the interview on Lynx, but if it on register I can take just some minutes and complete that client. So you would find that in a day maybe I can just counsel two or three just on Lynx, maybe two

**Researcher**: Ok that makes sense and how about for you

**Participant B:** I think the part is on the entries that we make on the information that is supposed to be handled just on a particular client just here on Lynx it takes much time as compared to the register so you may find that in a day maybe we have 40 cases so it can take us maybe 20 to 30 minutes to enter in the register but if you were to enter the same information on the Lynx it can take you much more that because some times that could also not be entered on the Lynx, but also the network. I think those are the challenges

**Researcher**: So how could we improve it to make it easier to capture,

**Participant B**: I think if at all now the issues of the internet, if at all it was like we do the test we capture, we do the test we capture it , but my dairy would say that most of our activities are in the community and usually those communities are where go and offer our services, most of those communities that we offer our services to internet has proved to be a challenge so we need to just like as we say after the work we come even if we entered from here. So again we here but we do enter… it would help us all, the protection that are suppose to be put on Lynx and also if our tablets are consistently working our system is consistently working we can use it would also help us. It works for a month and it stops. Yes but for the tablets that we are using…

**Researcher**: Sure?

**Participant B:** Yes but I don’t know for the tablets that we are using that is the issue even now as I am speaking they not working, it is even useless to asked for help ( inaudible) sorry

**Researcher**: Its fine, so you were saying basically the network and reliability because sometimes there would be issues and it would take a while to get addressed is there another one that would

**Participant B:** inaudible

**Researcher**: Ok, I will come to you in just a sec, but previously you have said that you do like all of the questions but it does also take a lot of time so (inaudible) Maybe you would still have the questions that good information but would not take as much time that you can think of

**Participant A:** Come again

**Researcher**: I am trying to see if previously you have said that you don’t want to remove the questions but then you also said that it takes a lot of time because it has a lot of questions. So I just want to check again if you think any of the questions could be removed or you still think, even if you said that it takes time but you want all of the questions there

**Participant B:** I think even though it take most of our time but those questions useful for us to provide quality services to the so I think on our part if we just the system is there on the system but the network there I think is good it, removing them it would compromise much of our service because we need as much as possible information to gather from the client for us to provide quality services if we scrap off some then again it means we won’t be able to probe more keep the data base for the clients we are able to provide the service

**Researcher**: And then I ask the same question to you if you could make it easier, if you had an idea to make it easier to capture on Lynx whether in the field or whether in the hospital would you….what would happen to make it easier or better

**Participant A:** I think as we have said the network maybe it would be better if I am in the field, it also takes time to capture if I come here and start reporting on the person that I was with in the field. if I am not working I could secure a certain time and start reporting on the same person I was with in the field so…I think the only thing that can help us is if we are in the field and the network is available it’s very easy to work on it to report on Lynx, I think

**Researcher**: Ok

**Participant A:** Like the only challenge is network because our reporting on Lynx take more time to complete, because you can start working and before you finish the network is out and you start again you come and you start at the beginning and you start reporting on the same person

**Researcher**: And when you are reporting on a person do you normally do it when the client is there, or do you record on a piece of paper and do it on Lynx when you come back?

**Participant A:** Maybe it can help us

**Researcher**: Which way do you normally do it?

**Participant A:** What?

**Researcher**: When you normally…

**Participant A**: In the field?

**Researcher**: Yes in the field do you bring the client and put it in the tablet with the client or do you use a piece of paper and the put it in the tablet when you get back?

**Participant A:** Again at the location, because in the field where I find the client is where the location show that I am in the field but when I come here it shows that that client is in the facility

**Researcher**: Yes that’s true

**Participant A:** That’s the only problem

**Researcher**: Ok, no it’s true. And for you

**Participant B:** Usually that is done in the community where you would find that client you start having any physical document (inaudible) you do now your capturing you can also capture together. So for an example the case came out positive you should be able to say this place is giving us more positive we should concentrate much in our file

**Researcher**: Sure, and then I just have one more question and then we are done. Do you have any final comments anything else to say about Lynx, what you like you don’t like, do you want to fix anything in addition to what you said is there anything else you want to bring up, I will ask you first.

**Participant A:** The challenge of Lynx, when the Lynx stops working we just report and we start waiting for someone to come also is a challenge if it’s someone within who know how to work one it can help us or if something is wrong he just work on it

**Researcher**: Fix it here?

**Participant A**: Yes

**Researcher**: Ok, it makes sense

**Participant B:** Yes I think I would agree with him if there was someone local to work on the issue that can develop by the system, it would be easier for us to continue using the service, and again if at all there was a way that if you are using Lynx the suggestion cause some register can be ( inaudible) double capture which is more of time consuming in that way maybe Lynx could be consistently if we just use it that data are able to extract it from there of the same client

**Researcher**: Ok anything else are we ok?

**Participant B**: No I am ok.
