# Supplementary material for: Health worker acceptability of an HIV testing mobile health application within a rural Zambian HIV treatment programme
Source: PLoS One. 2025 Jun 5;20(6):e0312646. doi: 10.1371/journal.pone.0312646 (PMC12140264; doi:10.1371/journal.pone.0312646)
Supplement: S10 File — (ZIP) [file pone.0312646.s010.zip › Transcript_6_deidentified.docx]

**Researcher**: So just as an ice breaker to start us talking, can we just quickly one more time say our names and how long you have been working as a lay counsellor or with Right-to-care, so just one more time to start I am Andries and I have been with Right-to-care for about five years , we can start this side

**Participant** A: My name is XXX I have worked with Right-to-care for about X years

**Participant B**: My name is X (inaudible)I have worked with Right-to-care for X years and some months

**Researcher**: Cool, Thank you

**Participant C:** XX I have been with Right-to-care since XX

**Researcher**: Ok

**Participant D**: I am ( inaudible) I have worked with Right-to-care from XX

**Participant E:** I am XX I have worked with Right-to-care for XX now

**Participant F:** My name is XX (inaudible) I have been with Right-to-care for X

**Participant G**: I am XX ( inaudible) I have worked with Right-to-care starting XX

**Researcher**: Ok thank you. So then the first question I have on Lynx basically is am going to be asking about how useful you think it is as an application how easy is it to use and how well does it fit into the working environment here, so firstly will be talking about how easier it is to use, how useful do you think it is and how easy it fits working at XX. So my first question maybe let’s start this side can you tell me your experience with Lynx such as

**Participant G:** Lynx I think it’s the easiest for me especially for tracking and to record those who are long distance ok, and for testing. When I test someone, I have to record and instead of written I have to record in the Lynx tablet

**Researcher**: And can I ask what is the difference between writing and versus using Lynx

**Participant G:** Its easiest way using Lynx instead of writing in the register

**Researcher**: And why is it easier?

**Participant G**: Huh?

**Researcher**: Why is it easier?

**Participant G:** If you can see we have a lot work to do at the facility we can’t be write in counseling at the same time using Lynx, so the easiest when you start counseling somebody you have to start using the Lynx

**Researcher**: Ok

**Participant G:** Yes

**Researcher**: And can I come to you next?

**Participant F**: Yes

**Researcher**: Have you had a similar experience or a different one, is it also easier to, is it easier to use Lynx than write or is it easier to use the register?

**Participant F:** Even for me it’s easier to use the Lynx

**Researcher**: Ok, and XX

**Participant E:** I think as they have supported one it’s easier for communication, because we barely get communication the network is at field and the health workers. Secondly it’s also an immediate communication between the client and myself as a counsellor because immediately I am asking questions because there are questions that are there immediately I am entering and that information is linked to the network other than writing because you write and then you start posting, the other thing that I have seen its quite easier actually its not a complicated program to use the Lynx

**Researcher**: Ok, and you said you ask questions and you put the answers in the tablet?

**Participant E:** Yes

**Researcher**: Which questions specifically?

**Participant E**: There are questions which we ask about for an example the age of the client have you tested for HIV, when did you get tested HIV, have you ever stayed with someone with TB and you ask also whether that person has got emotional (inaudible) and you ask also whether that person has got an operation

**Researcher**: Ok, so it’s all of them the pre counselling questions

**Participant E:** Yes

**Participant** **F:** Screening questions

**Participant E:** Yes screening questions

**Researcher**: Ok thank you,

**Participant G**: Unfortunately mine is similar to what XX (inaudible) has said

**Researcher**: Have you guys had a different experience from your colleagues, any of you? Like so far what I have heard is that it has made counselling a little bit easier in recording the patient information its made those go smoothly because you have that connection to the internet, do you have anything else to add on the general using of the Lynx, what you think about it and how do feel about it

**Participant** **B**: I think the information which is there at the moment is sufficient enough

**Researcher**: Ok

**Participant** **B**: Yes, unless otherwise my colleague have something that could be different if they think that there could be some additional than that, otherwise the information that is there at the moment is enough

**Participant B**: The one challenge sometimes we have is the talk time

**Researcher**: The data bundles?

**Participant G:** Yes the bundles yes

**Participant B**: Yes the bundles doesn’t come on time

**Researcher**: Ok, and is there, do you have good coverage in the out skates

**Participant C**: Yes we do

**Researcher**: And do you have good coverage when you go to the community?

**Participant All:** Yes

**Researcher**: Ok so that is good, so as long as you have network you are ok

**Participant A:** But there is some sites where there is no network so it’s a challenge for us to record using the Lynx

**Researcher**: Ok, when there is no network?

**Participant A**: Yes

**Participant G:** We had…

**Participant B:** On the MTN networks, some (inaudible) only have MTN network for data, they don’t have MTN network for data, they don’t have MTN network for communication so if you use MTN it’s a challenge for us

**Researcher**: Ok, and I haven’t heard from you Sir, how was your experience with Lynx?

**Participant C:** My experience in using the Lynx is mmm my colleagues have said everything

**Researcher**: Ok, don’t worry we have a few more questions also, we spoke a little bit on, but can you also tell me specifically what is difference between when you write information down by hand on the register versus on the tablet in terms of which is easier which requires more time, yes things like that

**Participant A:** I think by using Lynx it requires more time than the register you see

**Researcher**: Ok

**Participant A**: Because when you are documenting lets say you are taking the phone numbers it takes time rather than writing in the registers

**Researcher**: Because of having to type, type, type?

**Participant A**: Yes

**Participant F**: By the time this Lynx thing our boss there told us that we should record using Lynx when you are counselling or testing somebody, when you ask a question then you type, for an example when we were in the ward you are counselling and testing that’s when you can use Lynx, so we don’t know we still doing the same thing

**Researcher**: Are you using both Lynx and the register for counselling and testing?

**Participant** All: Yes

**Participant B:** We use both

**Participant A:** The only difference is that the we don’t have register especially when we are in the field so it saves time for use Lynx in communities when we come back from the community that’s when we enter the information into the register in the community, but when you enter the information the data is already gone

**Participant F:** But here at the facility we use Lynx when we are in the ward you are testing somebody that is when we are using Lynx

**Researcher**: Ok, so do you use it more in the facility or in the community?

**Participants All:** We use both

**Researcher**: You use both?

**Participants All:** Yes

**Researcher**: Ok, so I am coming to you to ask what is the hardest part for you to complete the capturing on Lynx

**Participant C:** What is the….

**Researcher**: The most challenging part, the hardest part , the worst part

**Participant C:** The most, the worst part no there are no difficult part

**Researcher**: One at a time sorry I will say the question again, so what is the most…what takes the most time with Lynx, what is the most challenging part for using Lynx, I will start here and I will come to you next

**Participant C:** I think we’ll the most challenging part is when we document this lot number, because sometime we went to the field and we don’t have that lot number because it’s a challenge to record that number, and also most of these clients they don’t know their (inaudible) number so it gives us a challenge to enter their (inaudible) number, some do have numbers some don’t have phone numbers so they are just not available

**Researcher**: Ok,

**Participant C:** And the next thing is the place when we are in the field, some places we don’t know exactly the name I can put on that day I am working from the….

**Participant B:**…location

**Participant B**: Yes, location sometimes we end up not knowing what location what name am I going to use where I am working from

**Researcher**: Ok, that makes sense

**Participant B:** And the areas where I find it’s a little bit difficult when I am doing the couple counselling because it is lengthy and when you are doing indexing it’s also lengthy, it takes a lot of time so that is the part which to me mostly challenging

**Participant G:** Even the network is the challenge

**Researcher**: The network?

**Participant G**: Yes, I don’t know if it’s the office set up, I don’t know maybe you can experience if you want to enter the information for the client you find in on the Lynx there is a red line so you can’t enter the client, I don’t know if it is you or…

**Researcher**: What do you mean the Lynx has a red line, like on the tablet?

**Participant G**: Yes on the tablet, on the Lynx

**Researcher**: Inside? Like I side the application?

(Inaudible)

**Participant G**: I am talking about the log in

**Researcher**: Oh ok

**Participant E**: I think what my friend has explained the only challenge that we have is time management, you know when you need to counsel a person and we need that information the client feels like you are delaying them, for us to the exactly that way but the client feels you are delaying them. For an example if you ask this question you ask this one you ask this one, and they feel you are wasting their time, that’s where it’s challenging

**Researcher**: Ok

**Participant E:** Yes that is the most challenging otherwise everything is all right

**Researcher:** Ok, thank you

**Participant G**: I think my friends have said what I needed to say so

**Participant F:** Yes so you can just go to another question

**Researcher**: Ok we are moving close to the end actually, we have two more questions, so for this one I want to hear what conditions or what settings would make it the most easy to capture on Lynx or the most hardest to capture Lynx, you kind of discussed it already that it is very hard in the community in a couple setting. For an example which day that you have that was very easy to capture on Lynx it was cool it was going nicely and then which day you didn’t capture Lynx at all because for some reason you were not able to or it was very hard. So I am trying to hear about the day that it was easy to use Lynx and the day that was easy to use Lynx and the day that it was hard to use Lynx

**Participant G:** Its easy to use Lynx…

(Inaudible)

**Researcher**: We will do one at a time

**Participant F**: I don’t know if shortage of….

**Participant G**: Test kits?

**Participant** F: No shortage of tests kits

**Researcher**: Its because of shortage of test kits?

**Participant F:** Yes

**Participant G**: Is it a challenge?

**Participant F:** It’s a challenge

**Participant A:** Because there is nothing you can enter if you don’t have a test kit

(Inaudible)

**Researcher**: Oh if you don’t have HIV test kits?

**Participant Al**l: Yes

**Participant F:** But (inaudible) so that it’s a shortage of test kits, especially (inaudible) there is a shortage of tests kits, so you may find that we maybe one week we don’t have test kits, so we can’t capture on the Lynx

**Researcher**: But also I have seen, I will come to you. I have seen I can see sometimes maybe we will say in January everybody working on Lynx all the time, but the I see in February not so many people only one or two tests come through, so how come some months its coming strong we have a lot of some months it’s coming strong and other months it looks like it’s forgotten

(Inaudible)

**Participant G:** It must have been at the hospital, and we have a lot to do sometimes you would find like for an example this one is working by himself at the ART department meaning CET so for us maybe we are forgetting something even today, or for one week and we have one person doing CET, so you are going to find that that one is the only one using the Lynx

**Participant C:** And also this one this happen to what she is saying maybe we are not in the ward there is a lot of patients whom I need to do, to test now that is a challenge for me to enter those clients in the Lynx because it take 45 minutes for me to complete a session because what we were told is that we should take 45 minutes for one clients, so for me I don’t know

(inaudible)

**Researcher**: I will come…XX has been waiting I will come to you

**Participant A:** There are two things that I can talk about, when we are in the field sometimes it become difficult for us to use the Lynx, depending on the type of work that we are doing into the field, for an example we are doing work on the device so we don’t normally do the work here, and the other point where I find a flaw, a single flaw there was a system maybe in the ladies from health previously there was just no system of dispensing class, so now they have improved on the system, they are saying they are giving them 3 months or 6 months drugs so after 6 months you don’t expect those people to come back and to after 6 months, so have a small number coming in at the hospital unlike previously when clients were just coming in randomly I think that we have also when…I think we have improved generally I can just say but there is no specific month were we can say in January we have a program like this it’s just it goes on the fore front there is no specific month were we say no this month we have a challenge with this one

**Researcher**: Ok

**Participant B:** I just want to add to what my colleagues said, he said that there are some people you find that in January you did well using the Lynx and February you did maybe nothing like what he said you would find that I can give an example in the last year starting from June, June up to July we had an exercise of making the files and doing ( inaudible) so in June July in fact I can say in two months and we didn’t do any work in testing so that we can report on Lynx. So that was a challenge when you see someone this month you are doing well then the next month test only to enter just two that is the challenge we face

**Researcher**: And do you think you only tested two people or you only had time to fill in two people but you tested maybe more

**Participant B:** No maybe you just managed to do two people, while you are assigned to other things

**Researcher**: Oh ok, did you want to say some things

**Participant C**: Just to top up to what he just said the challenges that he has just mentioned like today for an example we went outside to do home deliveries which will not be possible for us to do testing and previously we were putting files on holders it took us s good number of weeks before we completed and we were not actually doing any testing meaning we are not sending on the Lynx, so such thing would makes us slow down on the numbers that we send to Lynx

**Participant B**: What we are trying to say is that right now the minister of health has brought in the nurse who are doing the testing especially in the hospital here so we are not doing…for us now we are concentrating on communities, secondly when we are not testing as indicated you can’t do any tests that the what I think some of these some of the problem …

**Participant G:** But in the field we do tests

**Participant B:** It depends on when we have or when we don’t have, when we have…

**Participant F:** Test kits

**Participant B:** Test kits we do, but in most cases we don’t have the medicine that is though sometimes it takes us almost one month without any test kits

**Researcher**: Ok

**Participant F:** But what he is saying is true now the field our work is to do testing, it’s just that some are too emotional while we are busy doing testing

**Participant G:** Especially in the Lynx, when we test somebody we do go in the field and test, is it not true

**Participant F:** Yes we do test

**Researcher**: No I mean I do see it coming I just wanted you to explain why sometimes I see lots sometimes I don’t

**Participant G:** Campaign for TBT….

**Participant B:** And I was saying we need to emphasize on this one also we have a lot of indicators today we concentrate on this indicator the other day, tomorrow they will change they will tell you do this, the other day they tell you to do something else so you find there are these desperations we do

**Researcher**: No I understand, ok I will move to the next one, which is actually the last question. So what would you suggest to make it easier to use Lynx and I am asking this in terms of, maybe something on the tablet or maybe something for your work program in terms of your responsibilities so you can think of responsibilities or the way that the hospital is set up and the way the tablet is set up potentially a way to improve, some how either to be able to make it easier to do to capture on Lynx. Does that make sense or did I say too many words? So basically we have listed some challenges around network, sometimes test kits, sometimes you have to jump from indicator to indicator so what’s something that could make it easy thinking about all these things

**Participant C:** Maybe they can just come and make timetable, for us we don’t have a time table where by every day we do some tests and not because it is easier for us to use it but so we are going to come up with time tables, where by three of us will

**Participant A**: Can I say something?

**Researcher**: Yes

**Participant A**: Like she is saying sometimes we face a challenge doing our work because of the pressure because of other indicators, why can’t we have maybe in a week we submit the information weekly, maybe Tuesday or Wednesday maybe twice in a week other that everyday I don’t know that is our suggestion

**Participant C:** How could that be because the positive clients come at…

**Participant A**: If you are going to see a client on Monday I will keep the record and the if it is the actual day to report I report, because I know some information is reported not on daily basis there are certain days maybe on weekly, monthly

**Researcher**: That’s true that is still a good idea to make it easier with you indicators if to have more focused time kind of the same to as what you probably were saying and the issue with Lynx is that it is often if it is used fully you can see what time of day some one is coming in and then you can also see the location because I don’t know if you saw them here but there was someone was making these Maps, have you ever seen Maps?

**Participant F:** Maps?

**Researcher**: Maps, those were made as soon as you submit on you tablet we see the location if you submit all of your work here the map will show that all of work here and if you submit while you find someone in the community then it will show exactly where that person was. So it works best when you do it where you work, that when you come back so it will show that you worked here, but it makes sense

**Participant A:** What he is saying is trying to explain what I was trying to say the benefits of us using the tablet in the community, it will show where we are the location where you worked rather than that is what we do he was just trying to explain when you use the tablet for one week it won’t show from where (inaudible)

**Participant G:** I think we should just continue with what we are doing because we have seen so what we are doing everything shows,

**Participant A:** It shows

**Participant G:** It proves ourselves, I think everyday I think I should make sure that I add one or two, or five…

**Participant F:** For that we can’t promise that everyday you will need some supervision and you will always be directed to do, like you are to work on this one you are going to work on this indicator or this indicator so we can’t promise that we are going to do everyday, you can promise and fail to keep your promise

(Inaudible)

**Researcher**: I also wanted to check if you could change something with using Lynx itself to make it easier, what would you tell Right-to-care to make it easier for you? If you had something that you could improve

(Inaudible)

**Researcher**: It is the last question so once you…

**Participant C:** I think that we don’t have lots of challenges except maybe the dedication of duties those assignments we don’t have anything to report the other you are doing g something else that’s were we….

**Participant B:** Like what we said earlier on, we said they are assigning other people to go to the wards, to do the wards

**Participant G:** The testing

**Participant B:** The testing where by now the people who have been sent to do the Lynx are not there reporting is not easy in that way so if we discuss we talk to our supervisors so that we see a way in which we can go there to do the testing and so forth, the chances are give to us, like today no one was reported on the Lynx, and we are the only people that do the reporting here at XX with Lynx.

(Inaudible)

**Researcher**: what did you say if two people talk at the same time I am struggling to focus on which one, I heard what you said but I didn’t hear what you said

**Participant G:** No we are going to sit together and discuss how we are going to work especially using Lynx

**Researcher**: With whom?

**Participant G:** With our colleagues and our supervisors yes

**Researcher**: Ok,

**Participant B:** Maybe the improvement you will see on the Lynx, the Lynx are centralized on particular indicator. For an example city, and I would not suggest that other indicators also be focused here , so that instead of reporting this someone today, but I will do another indicator. But other indicators that are from Right-to-care hub they should be put on the Lynx, apart from PEP, PreP and the (inaudible) but other indicators that we do like the community delivery because they are also important, condom testing they are also important self testing its there also, because Lynx will take that information which is not part of this be included in the tablet so that we start reporting everything

**Researcher**: Oh ok that makes sense and I think that is a good suggestion, I made a note of it it’s here I will write it down, does anyone have anything else

**Participant G:** No

**Researcher**: Any final comments?

**Participant C**: We have learned something from this program

**Researcher**: The other programs?

**Participant C:** From this one

**Participant B:** These Lynx you have given us have no cover there is no safety we need covers (inaudible)

**Researcher**: What is the problem

**Participant B:** No covers

**Researcher**: I mean it looks like a nice tablet but you have to keep it safe,

**Participant B**: We need covers for Lynx so that it is protected

**Researcher** : Ok so it makes sense

**Participant G:** So what about talk time

**Researcher**: You want data and also to make sure that talk time is always covered and data

**Participant G**: Data is always there but talk time

**Researcher**: Talk time specifically?

**Participant G:** Yes

**Researcher**: But you need talk time for Lynx?

**Participant G**: For when you test somebody they become positive now you want to know the Parton we should communicate that that one has given the numbers so there is no talk time how are you going to communicate

**Researcher**: Sure that makes sense

**Participant A:** Sir we have only one tablet, what do you call that tablet?

**Participant C:** Call centre

**Participant A**: We have only call centre, now that one call centre is the one we use to communicate when we are in the field and the hospital they use the same call centre for an example I am in the field maybe 3 kilometres away from that person how do I link myself to that person, it is easier for us to have our own talk time like the professional counsellors they receive talk times on their phones, ok that is a challenge there also and you find that I keep calling on my phone, I can’t

**Participant C:** We can not call on the Lynx

**Participant B**: Yes they are blocked

**Participant G:** You are saying they are blocked?

**Researcher**: Huh?

**Participant G**: These tablets

**Researcher**: I don’t know

**Participant C:** We can’t use them

**Participant B**: We can have talk time for this ( inaudible)

**Researcher**: Ok, just to also be clear I am not in charge of that set type of rules because that is up to the Right-to-care provincial offices they set the rules for, because you are employees of the Right-to-care so I am employed by Right-to-care South Africa and this is Right-to-care Zambia. So I will give them my report from what I heard you say, and I will say that the testing will improve if you give them talk time, I mean as part of the report they will hear this but then I can not promise anything, but I will put it in the be reporting

**Participant D**: Yes

**Participant G**: We are also promising that we will using the Lynx and we are not going to stop its also part of our indicator special testing

**Participant B**: And it’s good that you have come and discuss with us its one way which will make us improve on that one because maybe a discussion one on one with you together one on one

**Participant C:** And we want other bosses to come and visit us from different departments is that you come for Lynx we want another one for tablets we tell them what’s our challenges yes we improve, so from we are saying thank you so we want another one from a different department to come

**Researcher**: I will pass the message
