# Supplementary material for: Health worker acceptability of an HIV testing mobile health application within a rural Zambian HIV treatment programme
Source: PLoS One. 2025 Jun 5;20(6):e0312646. doi: 10.1371/journal.pone.0312646 (PMC12140264; doi:10.1371/journal.pone.0312646)
Supplement: S10 File — (ZIP) [file pone.0312646.s010.zip › Transcript_7_deidentified.docx]

**Researcher**: Just to start us off I would like to go around and just say us how long you have been working at a hospital, whether its this hospital that you have always been at or another one I am just curios just to see how long have you been working with health care, can we start?

**Participant A:** This is my X year

**Researcher**: X?

**Participant** A: Yes

**Participant B:** I have been working for X years

**Researcher**: X years?

**Participant B:** Yes

**Researcher**: Ok

**Participant C:** X year

**Researcher**: Ok

**Participant D**: X years

**Researcher**: Ok,

**Participant E:** X years

**Researcher**: Ok

**Participant F:** More X years

**Researcher**: Ok

**Participant G**: More X years

**Participant H:** X years

**Researcher**: Ok, so I can see we have a good amount of experience. Can you tell me, Ok I will start again over here, can you tell me how it was like to use Lynx as a lay counsellor

**Participant A:** It was quiet a bit different though its more like an electronic screening tool, because before we would just use the hard copy as screening tools then we were given a table. For me since I was always based at the facility it was easy because of network, except for the counsellors that were going in the field. Yes we have a bit of a challenge because you find that they have to be there and it can be something where you test somebody and you record but then you find that sometimes you don’t have network, so it’s a bit of a challenge, but still interesting

**Researcher**: Ok, and for you?

**Participant A:** He hasn’t used, she hasn’t used so you will just have this one, the madam and the two gentleman there

**Researcher**: Ok, just I will probably focus my questions on the ones who have used, unless you have any opinions on Lynx, did you see your colleagues using it?

**Participant A**: No I think the time they joined us the tablets were down

**Researcher**: Ok

**Participant A:** Yes

**Researcher**: Ok, so can you tell me about your experiences using Lynx?

**Participant D**: uhh I am going to explain myself in my mother’s language

**Researcher**: Maybe if you could do the interpretation

**Participant A**: Do the interpretation…oh ok alright ****Bemba****

**Participant D:** ****Bemba****

**Participant A:** So sir he is saying the way he was using it once he test somebody he enters the information on the tablet and he does the immediate counseling

**Researcher**: Ok

**Participant A:** Yes so that is how he was using it ****Bemba****

**Participant D**: ****Bemba****

**Participant A** :He says at first it was ok but when we start having system challenges it was difficult to work with

**Researcher**: Ok, and madam?

**Participant E**: Just the same as me ***Bemba***

**Participant A**: So she has similar problems at first it was ok but since we are in a place where network was a bit of a challenge you find that you don’t send the report as you test so you take a bit of time to send it, you have done the testing in the morning but then you find sending the report a bit late, ok so same challenge as him at first it was ok but later on we start having system challenges

**Researcher**: Ok, I understand

**Participant D:** We have the same problem, same problem

**Researcher**: So you Sir, similar or different?

**Participant A:** No he never used

**Researcher**: Never used, ok can you tell me how, maybe when you were counseling or testing a client or recording any where in the counseling process how it was different using a table versus using just a just paper register

**Participant A**: Using a tablet was a bit of a challenge because then you wouldn’t have to pay attention to the client because you have to, as the client is talking to you just have to keep on entering what ever they are saying the information that they are giving to you, so you find that you don’t do much of the counseling because you have time limited and you don’t pay attention to what the client is saying because you have to you are supposed to pat attention so your time is divided, so it was a challenge when we have to do, because we told its supposed to be an instant thing, isn’t it? you test you also do the reporting on Lynx. So it was difficult because you talk to a client what ever information they are giving you, you also enter it. It used to hinder us in talking to the client on a personal level

**Researcher**: Ok

**Participant A:** Yes, it was the hard copy ones were good because then we would just write rather than you have to be…where we are as a set up the clients would find it a bit rude when you are on the phone they would not understand that it’s the application that I am using, yes so it was a bit difficult

**Researcher**: Ok, thank you

**Participant F:** The location

**Participant A:** That is what I am saying I am saying it’s the area, maybe in other places it might be different where these people are acquainted to electronics, but for here it’s kind of difficult it’s like you are on the phone you are not paying attention to the client (inaudible) hinder us in a way

**Researcher**: Ok, can I ask the same question to you Sir, if you noticed any difference when you were testing or counseling a client especially recording, the difference between when you were using your paper register versus the Lynx tablet

**Participant A**: ****Bemba****

**Participant D:** ****Bemba****

**Participant A:** So he is saying the Lynx application is better than the ones we used on the hard copy because those ones it’s not him who is going to see what he is doing and the other people that are working on the tablet or that are minding the application they are able to see what he is doing so he found its better because it’s just not him looking at his work

**Researcher**: Ok, sure thanks and from you too anything different

**Participant E:** ****Bemba****

**Participant A:** Ok so they are saying it’s more of proof that they are working

**Researcher**: Ok

**Participant A**: Yes unlike the hard copy because no one will see that they can do their work so it was better because then people are able to see that since the tablets came with a name attached so that they are able to see that (inaudible) is working at this particular time so she is saying it was better than the hard copies

**Researcher**: And for you Sir?

**Participant F:** Same, same

**Researcher**: Ok, so I hear that it can take time to use it and also sometimes maybe your client that you are counseling will be uncomfortable because of you using the tablet while you are counseling which could be a sensitive time and also you do like some parts of it so maybe while you were suing it how did you overcome the challenges when they were occurring or what happened

**Participant A**: So towards they end what we were doing we started to enter what ever we have worked through out the day and then we wouldn’t be doing the entering as we were doing g the counselling we would do the counselling get the details and enter them in a hard cover book and then at the end of the day when we come back To report that is when we do the reporting on the Lynx tablet

**Researcher**: Ok

**Participant A:** So even that also helped with network because they do community testing so you would find they are at a place where there is no network so they have to come at the centre where there is network and the entering so it was also one way that we over came the network challenge

**Researcher**: Ok, XX can you tell me some other challenges maybe that you face when using Lynx or is it mainly the ones that you have said so far

**Participant A:** The other challenge was that they used to get blocked most of the time, so you find you want to work on it being locked out of the system so now you have to start waiting for somebody to unlock you it used to make it a bit difficult . So you find that some information you have not reported some you have reported at the end of the day when compiling you find that what you have entered on the tablet it’s probably a few that what you actually…

**Researcher**: Ok, and that was mainly because it was locked or because of the time?

**Participant A:** No usually it’s just being locked out

**Researcher**: Ok, Sir can I also ask what challenges you faced besides network or the time

**Participant G**: Same

**Researcher**: Same?

**Participant G**: Yes

****Bemba****

**Participant A:** So tablets are quite big in size so we don’t have to carry because we have to walk quite long distances so if you have a bag to carry books and you have a big tablet and your phone so it was a bit cumbersome

**Researcher**: So it was carrying all the work with papers and all that

**Participant E**: Yes it was

**Researcher**: Yeah I know a bit about that, and I know they have different sizes now

**Participant A:** Maybe if we were given smaller phones it would be helpful

**Researcher**: Yes, and for you two where there any challenges that haven’t been mentioned yet?

**Participant F:** ****Bemba****

**Participant A**: So he says for him it broke because he was on a bike when he was riding it fell off

**Researcher**: And for you Sir?

****Bemba****

**Participant A**: No challenge

**Researcher**: Ok so you have already kind of talked about sometimes you wouldn’t submit as much one day or another day which we could also see from our side because sometime we could also see on a registers you have 30 people tested today but then on Lynx there was only maybe 15 or maybe 20, but then tomorrow or the next day we see now there are 30 people tested but there are 30 also on Lynx so all of them are there but then the next day maybe it’s down to 15 or 12 instead of the full 30. So can you describe more of the situations that would either allow you to have all of the tests in the Lynx and also why they would not have all of the tests in the Lynx

**Participant A:** Sir I experience are similar situations some time back when we were using them, you find that you have entered all the tests in the Lynx in the night only to find that actually only one test has been entered, we also used to have like that there were counsellors who would come from the field and say that no I have entered all the test but when I synchronizing it’s only showing one test that was recorded, yes so we use to have things like that counsellors who would come from the field they would say no I have already entered everything but when you synchronize their devices you only find two or one, so we didn’t understand why the system only registers one person yet you registered many. For my side it can show that you registered all of them but when you go to view my status it would show you a few. Yes so I am not sure if it’s just the system, and the other challenge that could have contributed to that is like I said earlier you find that where you are working from there is no network then you would come at the facility quite late you would have entered the register the hard copy registers and then you fail to enter on the Lynx tablet. So you find that you enter only half when you have done a few of them. The following day you would be working from the facility were there is no time to enter the most people that you have tested, so those are the two kind of challenges we had

**Researcher**: Can you translate the same questions to your colleagues

**Participant A**: ****Bemba****

**Participant E:** ****Bemba****

**Participant A:** So she is saying sometimes you find that you are entering quite right and then you have network challenge or you are locked out of the system

**Participant G**: Sometimes….

**Participant A**: So he is saying sometimes you probably come back later from the field so you thought you would enter because the distance are quite far so the are afraid that by the time they come they have to leave this morning, the same report, the registers and there are various registers

**Researcher**: Ok, it makes sense. So in terms of…it sounds like one of the main issues was the time it takes to complete it so potentially what would be a way…it’s sounds as though you could potentially shorten it

**Participant A:** Yes that could be really, really good because there a lot and lot of questions that we need to answer and inputting and it’s quite draining, because like a screen for tuberculosis its also in there family planning it’s also in there so it takes like…just for one client it takes like 20 to 30 minutes and then you have, like at the facility let’s say like on a normal busy days you see like 50 people and then if you are spending like 20 to 30 minutes on one person you find you won’t be able to do your work properly. So maybe if the questionnaire could be a bit shorter or it can be incorporated some of the questions could be made in one it could be very helpful

**Researcher**: Ok, can you think of another way to improve the application to make it easier to use either in the field, the community or facility

**Participant E:** ****Bemba****

**Participant A:** ****Bemba****

****Bemba****

**Participant A**: So he says there are a lot of repetitions on the questionnaire and then for a positive client it’s even worse because it’s like double the questions, yes so if maybe they can maybe like I said earlier incorporate some other aspect and put them in one, and they are also saying the is too long, because it’s just not the client information you still have to put the test kit information from the batch number to the expiring date ****Bemba**** for both, so it’s quite a lot

**Researcher**: Ok, any other

**Participant A:** No they almost said the same thing

**Participant H**: ****Bemba****

**Participant A**: Ok so she is our clinicians

**Researcher**: Ok

**Participant A:** She says, suggests that maybe another way it could work is make the system off line user friendly, yes where you just don’t have to rely on internet were you are able to enter the information you want offline and when you are in a place where there is network work you can easily just switch on

**Researcher**: Ok, makes sense. Can I also ask was the last time the tablets were working in this facility?

**Participant A:** Maybe when they were just properly working towards the end of 2019

**Researcher**: Oh so it’s quiet some time

**Participant** **A**: 2020 we haven’t used them, because for him it’s blocking (inaudible), mine the network has gone I can’t use it it’s just there, for her the internet it shows just the system doesn’t allow her in, and this one it doesn’t have internet and even her it’s quite the same, the problem is with the tablets you would find that today it would work tomorrow it won’t you are just plug out

**Researcher**: Ok, I ask because some of the things you describe there is a point last year were we met with several of the counsellors especially the HTS coordinators and we did shorten some of the requirements for Lynx we moved the lock number and a few of the things, so…

**Participant A:** We haven’t used (inaudible)

**Researcher**: That is what I was referring to that your suggestions are good and we have heard them also from other facilities

**Participant A:** We haven’t use them from the time you upgraded it

**Researcher**: Yes that must be it, ok because we are always making upgrades we will also take still what you are saying now so the next time we can make the update it can also still be improved again to improve the offline function…that’s not what u am supposed to be here talking about. So if you wanted to use Lynx more during the day would it affect the way that you are working or also the other way around is do your work responsibilities affect how much time you have to use Lynx?

**Participant A**: Yes

**Researcher**: Can you give an example?

**Participant A:** Ok we have other indicators that we also help work on, so it’s just don’t limit to just testing. So you find that you have to visit a client for adherence support and you have to probably check on a client that has missed an appointment for drug pickup, so you find that if you spend much time maybe on testing and maybe entering on the Lynx tablet you won’t be able to do this other works

**Researcher**: Right

**Participant A**: Is it the same or? And also the other one is the number of clients that you see there are days that you have a lot of clients and there are day where you a fewer clients, depending on the schedule for the clinic. So like for an example here we have Tuesdays, Wednesdays and Thursdays which are quite busy so it would be really impossible for us to work on the Lynx and complete our work.

**Researcher**: Ok, any other comments

**Participant E:** ****Bemba****

**Participant A:** So it wasn’t effective for everybody, so they still want the phones not the ones that we currently have, because they are not functioning properly so they are looking forward to different phones that they can still do the Lynx work

**Researcher**: Ok, I am hearing a little bit of both that it works but it also doesn’t work

**Participant A:** No, no not that it doesn’t work the way it was, like you said it’s been upgraded its been shortened it will just work. It’s just the timing maybe if it’s an offline user friendly where by you do the work even in an area where there is no network or I can do it after I am done with my work if I have a busy schedule, I am done with the work I get proper information then I do my Lynx later

**Researcher**: Interesting

**Participant A:** It can work. But initially when they came we were told it was supposed to be an instant kind of thing, where am with a client the moment I test the client I enter so now if I have 50 clients waiting for me outside then I am spending 30 minutes with one client it is why we were saying it would not work. But if we are being told that it is possible shortened and we can work off line then that’s ok

**Researcher**: Ok, it’s cool. Ok I think we have touched on most of it can you maybe just tell me one more time and talk a little just more on the differences of using it versus in the community versus in the facility, if you use it in the community or if you do not

**Participant A:** ****Bemba****

**Participant H:** ****Bemba****

**Participant G:** ****Bemba****

**Participant D:** ****Bemba****

**Participant A:** So the difference mostly with the community and the facility is that in the community we don’t usually find somebody sitter waiting for the testing we usually do door to door where we find somebody to test we go to them and they test, so you are not seated. You find that here you spend maybe 5 minutes with the person you are testing, he leaves another 10 minutes so you don’t have a place where you can properly work on the Lynx, unlike the facility, because at the facility obviously I will have a room where each client with privacy I am sitter and the client is sitter comfortable as I enter, for the community its different, so like he said like the community its also distance. You find that you have clients that you want to go see, one comes from a different village and the other comes from a different village so you have to ride from one village to another so it time consuming, so you don’t want to spend much of your time on a client because you also want to reach as many clients as you can, so if you are sitter on one client for too long so you cut short on the others that you wanted to see on that particular day,

**Researcher**: Ok

**Participant A:** Yes so they said quiet the same thing, distance there is no much of privacy in the community, and just the comfortability of you being sitter and enter you work

**Researcher**: Ok, and with potential…I am just guessing but would a smaller tablet its again more easy to bring and a shorter time to complete would that assist with those challenges?

**Participant A:** It would, it would definitely assist because by then you would use just one hand, because the tablet you have like to use both hands and then you are there but a smaller phone can work and a shorter screening tool on it, and offline

**Researcher**: And offline of cause. So those were all of my main questions I wanted to go through but then if you have any other final comments on Lynx especially just anything you want to include in this report because a lot of people can look in it so don’t be shy feel free to say any ideas or comments

**Participant E:** ****Bemba****

**Participant A**: ****Bemba****

**Researcher**: She is saying you must be well equipped

**Participant A:** Yes

**Participant B/C**: ****Bemba****

**Participant A:** Ok so the two that have never used are saying that they are eager and really want to know how the program works and the are looking forward on the invitation once it’s started. And then the one that already used they are just saying the shorter the screening tools and the smaller the phones the effective the work. Yes but the two that haven’t used are eager to…they are looking forward to use it, so the looking forward. I think for my comments it was a good initiative where we were able to do the work and instantly report because sometimes you find that people have reported for work and you find you get sitter the whole day so in that way it was able to monitor our work, yes and actually see if we are using the right equipment are we using date that has not expired, because you find that you enter a batch number that has expired it won’t allow you to do the test, so in that way it used to monitor our work because you hear stories that somebody used a determine testkit that had expired, so if the Lynx can be brought back in full force its quite helpful because it also monitors our work

**Researcher**: Ok

**Participant F:** And it would make the work easier

**Participant A**: And reporting huh, reporting would be easier because we have to count, if you do the work you count how many you have tested. But then with the Lynx you just enter every body and when it synchronizing it tells you how many

**Researcher**: Ok, thank you, I don’t know if any body has any thing else

**Participant D:** ***Bemba***

**Participant A:** Oh so they thought you came with the phones

**Researcher**: Oh sorry I came with questions
