# Supplementary material for: Health worker acceptability of an HIV testing mobile health application within a rural Zambian HIV treatment programme
Source: PLoS One. 2025 Jun 5;20(6):e0312646. doi: 10.1371/journal.pone.0312646 (PMC12140264; doi:10.1371/journal.pone.0312646)
Supplement: S10 File — (ZIP) [file pone.0312646.s010.zip › Transcript_8_deidentified.docx]

**Researcher**: First can you tell me how long you have been working at the hospital?

**Participant A:** Almost X years

**Researcher**: X years?

**Participant A:** Yes

**Researcher**: OK always with this hospital?

**Participant A:** Yes

**Researcher**: Ok, that’s a long time

**Participant A**: ( laughs) It’s a long time

**Researcher**: And how well has it been to use Lynx while you were working as a counselor?

**Participant B**: ***Bemba***

**Participant A:** Yes

**Participant B:** What she is saying is that it was easier

**Researcher**: Easier?

**Participant B**: Yes

**Researcher**: OK. Can you tell me especially how it affected the way you would be testing or counseling or recording, so just the way you are doing your work? How it affected it

**Participant A:** ****Bemba****

**Participant B**: She is saying it was helpful because reporting was easier, once you finish you counseling your testing immediately you enter the Lynx then your report go

**Researcher**: Ok, that is good, and how much time did it take to complete Lynx while you are counseling?

**Participant A**: After ****Bemba****

**Participant B:** ****Bemba****

**Participant A:** *** *Bemba****Just a minute

**Researcher**: OK, so for you it was very easy?

**Participant A**: Yes

**Researcher**: OK, can you then maybe tell me some of the challenges that you faced

**Participant B**: ****Bemba****

**Participant A:** ****Bemba****

**Participant B:** What she is saying is that the phones that you have brought them had problems

**Researcher**: Ok, and would freeze and things like that?

**Participant B**: Yes they would freeze

**Researcher**: OK, were there any other challenges as well

**Participant B:******Bemba****

**Participant A:** ****Bemba****

**Participant B**: We never had adequate training on how to handle because it was one day, training some of us are not conversant with these smart phones and its not easy

**Researcher**: OK, sure

**Participant B**: Some of the applications we never used them before

**Researcher**: OK, it all make sense

**Participant B**: So it was like joburg training? Joburg training? We gathered a lot because….

**Researcher** : They are going to train…

**Participant B:** No we did not train, I think that was the biggest challenge more especially with me because I am not too conversant with smart phones so some applications where difficult to use

**Researcher**: Were your colleagues able to assist you or did anyone assist like a follow up training or were you eventually able to learn to use it or was in unusable?

**Participant B**: It was not up to the expectations not with me

**Researcher**: OK

**Participant B**: With me

**Researcher**: OK, can I ask for you

**Participant B**:***Bemba***

**Participant A**:***Bemba***

**Participant B:** What she is saying is, if she could only use it for reporting purposes not all applications

**Researcher**: OK

**Participant B:** Yes sending reports was easier but using other applications was a challenge

**Researcher**: Ok, which other applications?

**Participant B**:****Bemba****

**Participant A:******Bemba****

**Participant B:** What she is saying is that, once you error it wasn’t easy to get back and finish all your business. Unless you have some body who is conversant

**Researcher**: OK I hear you

**Participant B**: And the other challenge…because we could go there with the Lynx phone because we did community work, you are alone there and then the other person who is conversant with the phone is very far away then it was a bit challenge, but what we can ask for to over come these challenges is adequate training maybe for 5 days just but it was just for one day not even a day for some hours

**Participant A**: for some hours

**Participant B**: No go do this ( inaudible)

**Researcher**: And can you tell me more about the difference of using the Lynx tablet versus writing on the paper register for counseling and also recording your clients, the differences you would see when you are just writing on paper versus…

**Participant B**: I think I can answer you this question

**Researcher**: Yes

**Participant B**: You know paper work is tedious, I think this time around we have made record using these smart phone is easier but the challenge we had is how to operate it

**Researcher**: OK

**Participant B:** Its not …… because paper work is tedious and using paper work you can easily lose the information

**Researcher**: Sure you can leave it somewhere

**Participant B:** Or even the paper is destroyed, using the computers information is restored

**Researcher**: And for you anything different?

**Participant A:** No

**Researcher**: It’s the same?

**Participant A**: The same yes

**Researcher**: And can you maybe tell me the difference with using the tablet in the community versus the facility you said in the community it’s a bit more challenging because you cannot ask your colleagues maybe to assist…

**Participant A**: Yes

**Researcher**: Buy is there other difference between using it in the community versus the facility

**Participant A:** No

**Researcher**: It’s the same?

**Participant A**: It’s the same

**Participant B**: It’s the same as long as you are conversant

**Researcher**: So is it easier then to use it in the community or in the facility

**Participant A**: Both just both

**Participant B:** It’s the same

**Researcher**: OK…

**Participant B**: Because you must understand when you are in the community it would even show the location where you are

**Researcher**: Exactly yes and then I will ask this one for you because you have even used it more but we can se sometimes the Lynx submission they come in they can be maybe 20 so each week maybe on the registers there is maybe 20 tests we can see on Lynx some time we have a the work from the registers also in Lynx but maybe the next week there is some tests missing from Lynx that are in the registers but not on Lynx the maybe the next week it’s

**Participant A:** There is some, yes

**Researcher**: Acxatly can you maybe describe an example why sometimes they were less and sometimes all of them where her

**Participant A:** ****Bemba****

**Participant B**: What she is as she alluded earlier on that the biggest challenge she had is when she had with Lynx when it freezes of course you won’t continue using it because there is no other person to help you restart, I think that is why in the register you had more numbers than the , because when it’s frozen you couldn’t work

**Researcher**: And when it freezes is there someone here who can assist or does it unfreeze by its self or what happens once it freeze or is the tablet stuck

**Participant A**: It stops working, even it can stop the whole day without working

**Participant B**: What she is saying is that unless somebody who is conversant is around

**Researcher**: So sometime maybe another co-worker could come and help unfreeze but maybe if you were trained on how to fix

**Participant A:** No problem

**Participant B:** And you know in our culture how it is when you know something you become adamant when the other person asks for help

**Researcher**: Sure

**Participant A:******Bemba****

**Participant B:** But once you when you have adequate training we promise you

**Researcher**: Ok, that make sense. So I hear about the training and the tablet sometimes freezing

**Participant A:** Yes

**Researcher**: But can you think of something else that could be done from our side to make it easier to use the tablet, something else to make it easier to do capturing or improve it for you

**Participant B:** Just the training

**Participant A**: Just the training

**Researcher**: What else can be done, is there anything more

**Participant A**:****Bemba****

**Participant B:** Maybe you can change…

**Researcher**: The tablet?

**Participant B**: Yes

**Researcher**: To change the tablet as well?

**Participant A**: Yes

**Researcher**: And for you what would you want in a different tablet like

**Participant A**: ****Bemba****

**Participant B**: Simple smart phones

**Participant A**: Simple like this one

**Researcher**: A simpler device?

**Participant A**: Yes

**Researcher**: Is network a challenge her or are you covered

**Participant A**: No

**Researcher**: You are covered in network

**Participant B:** We are covered

**Researcher**: OK so at least that is nice

**Participant B:** We are covered

**Researcher**: OK, we have actually been quiet quick, can you think when you were doing your capturing what sort of difference did you notice during the day when you were using if it was a busy day versus a quiet day

**Participant B**: Come again

**Participant A**: ****Bemba****

**Researcher**: How was it different to use Lynx if the hospital was busy versus if the hospital was quiet

**Participant B:** Oh yes, when the hospital was busy you sport a challenge

**Researcher**: Can you explain more beside the same reasons that you have is there a different reason? What reason?

**Participant B:** When the hospital is busy?

**Researcher**: Yes

**Participant A**: ****Bemba****

**Participant B**: What she is saying is since we are main the hospital is quite busy we can share the work when one is entering on the Lynx the other one is with the client so that we don’t waist time for our clients

**Researcher**: OK, and how about when it’s more quiet

**Participant B**: No it was easier it is fine

**Participant A:** ****Bemba****

**Researcher**: OK that is all my questions is the any other like if you could say something to the bosses about Lynx to make it better we hear you are asking for the training and a different tablet

**Participant A**: To be given new phones, Yes

**Researcher**: Anything else?

**Participant B:** No I think we are done, all we need is adequate training

**Researcher**: And the same for you anything different?

**Participant A**: The same please

**Researcher**: OK that’s it thank you
