# Supplementary material for: Health worker acceptability of an HIV testing mobile health application within a rural Zambian HIV treatment programme
Source: PLoS One. 2025 Jun 5;20(6):e0312646. doi: 10.1371/journal.pone.0312646 (PMC12140264; doi:10.1371/journal.pone.0312646)
Supplement: S10 File — (ZIP) [file pone.0312646.s010.zip › Transcript_9_deidentified.docx]

**Researcher**: OK just to start us of, can you tell me how long you have been working in health care

**Participant A**: It been more than XX, yes

**Researcher:** Always in XX?

**Participant A:** No not in XX, in XX. In XX I stayed for almost XX years.

**Researcher**: OK, that’s nice. And can you tell me about your experience of using Lynx at XX?

**Participant A:** About using Lynx? It was just fine experienced it was fine, yes

**Researcher**: Is it working right now or is it having issues

**Participant A**: No right now it’s not working

**Researcher**: OK, so when it was working how did it affect the way you worked

**Participant A**: It wasn’t affecting anything

**Researcher**: Would you maybe use it differently if…could you maybe tell me the difference if you would test a client or capture a client when you are doing it on paper versus when you are doing it on Lynx?

**Participant** A: Ok, it was easier using the Lynx when you are in the field, it was easy for the reports, you report and you enter afterwards so it was faster

**Researcher**: OK, why do you think it was faster?

**Participant A:** Because we are just…when you test you submit when you test you submit and you enter when you come after the field

**Researcher**: OK and how about when you are at the facility is it also easier at the facility or different?

**Participant A:** At the facility it was also easier because when you test from here you enter, you enter data from Lynx and in the register

**Researcher**: OK, You sounds as though it was easy to actually capture the person or do you struggle with the capturing

**Participant A**: No the was no struggle in entering the client

**Researcher**: Ok, that is good, was the any point in terms of using tablet where you would have challenges, or would have some thing hard

**Participant A**: Some times about the pin, it was not submit when you want to submit it was not going through, about the pin we don’t know what happened

**Researcher**: And the pin, this is the pin you use to log in to your account?

**Participant A:** Yes

**Researcher**: Has there been points where the pin didn’t work and the it was able to be fixed? Or once the pin stops working its finished

**Participant A:** No it was fixed some time back, but then again it stopped

**Researcher**: Ok, that makes sense. And then in terms of how much time it takes to put it in the Lynx versus to put it in the register, which one do you think…which one do you prefer to use

**Participant A:** As I had said the Lynx is faster and in the community

**Researcher**: OK

**Participant A:** Yes you captured all the details of the client

**Researcher**: But then can you tell me more about the difference between the community and the hospital, because I hear you when you say in the community you are able to capture quickly and when you get back you put it in the register. But when you are in the hospital when it’s a busy day and lots people are coming could it be different

**Participant A:** The only difference I could say is that when you are submitting the reports this one would not go faster than the Lynx

**Researcher**: OK so its still faster with the Lynx?

**Participant A**: Yes

**Researcher**: And we can see sometimes in different months even when Lynx is working that sometimes lots of people are submitting and we can see the numbers of submitted tests is very high almost the same as registers but other times even when Lynx is working we can see not so many people are submitting and but the numbers are there at the register

**Participant A**: ( inaudible)

**Researcher**: Its ok, I will say it again. Sometimes we can see even when Lynx is working that maybe not all of the same test that are in the register end up in Lynx some times we can see lots of people are submitting in Lynx and the numbers are high and other times we can see not as many people are submitting in Lynx and the numbers are a bit low. Do you know maybe why some times, some week the Lynx is getting submitted and other weeks are a and there I no right or wrong answer we just want to understand how to improve from our side

**Participant A**: ***Bemba *** can I say that I don’t know

**Researcher**: You don’t know?

**Participant A**: Yes,

**Researcher**: Do you think it has something to do with how busy the hospital is or maybe the different responsibilities that you would have, like maybe you are focused on a different indicator or maybe you would spend more time doing other activities or too busy at maybe something else

**Participant A**: Maybe it’s what we focus on is testing in the field, so maybe sometimes it’s like that focusing on other indicators but what we do mainly is testing

**Researcher**: Ok that’s fine so could you think of a day that would be easier to capture on Lynx and the day that it would harder to capture on Lynx, so maybe one day you are able to capture everyone nice and easy, but then there is another day where you don’t capture all of them that day and then you would have to maybe capture it later on

**Participant A:** If I capture later that means I won’t get all the details, we do enter it when you are with the client and not when you are not with the client

**Researcher**: Is there anytime…because we can see that sometimes the counsellors are unable to capture with the client and then they put it in later do you know, can you think of something that would cause that to happen? Whether it’s too busy or…

**Participant A:** That means you are busy with something like other indicators as I said

**Researcher**: OK and if you could think of something to improve Lynx or the way that its being supported for an example since we could see that the pin is locking you out maybe a way to improve it could be IT support so that there is someone who would be able to assist you more quickly if you get locked out . But you could also think of a way to improve that capture or anything else that would make it easier for you to capture more people?

**Participant A**: Come again

**Researcher**: Just any way to make it easier to put all of the clients into Lynx, besides fixing the pin issue more quickly

**Participant A**: Maybe you reduce the questions, there are more questions in the questionnaire ( laughs) Maybe reducing some questions it would be easier

**Researcher**: Ok well then it makes sense. Ok we are just about done. And so what do you like most about using the tablet? What is your favourite part when it’s working

**Participant A:** It’s easier to communicate with the ones remaining at the facility when you are in the field

**Researcher**: Ok, which ones remaining at the facility, the other counsellors or the other clients?

**Participant A**: The other counsellors we have the professional counsellor

**Researcher**: Ok that’s good and is there something that you like or don’t like about it when you are at the facility? I hear that you say it’s a bit long so I guess you don’t like this part, is there another part that could be made better

**Participant A:** Sometimes the clients get irritated when you ask them to much questions

**Researcher**: So the clients don’t like how many questions there are sometimes

**Participant A**: Yes, but not everyone

**Researcher**: Ok that makes sense, and if you have a client that doesn’t like all the questions what do you do from your side, do you, how do you overcome the challenge

**Participant A**: You try by all means to be faster

**Researcher**: OK, I think that’s about all of the questions, do you have any final comments?

**Participant A**: No I don’t have

**Researcher**: Cool all right.
