# Supplementary material for: Health worker acceptability of an HIV testing mobile health application within a rural Zambian HIV treatment programme
Source: PLoS One. 2025 Jun 5;20(6):e0312646. doi: 10.1371/journal.pone.0312646 (PMC12140264; doi:10.1371/journal.pone.0312646)
Supplement: S10 File — (ZIP) [file pone.0312646.s010.zip › Transcript_10_deidentified.docx]

**Researcher**: So I will be asking you some questions about Lynx it will be focusing on kind of three different part it will be how easy it is to use Lynx, it will be how useful to use Lynx for your work and how well does it fits into your work environment here. Just to kind of start us off can you tell me how long have you working as in health care

**Participant A:** With Right-to-care?

**Researcher**: Any hospital

**Participant A:** It’s 13 years

**Researcher**: 13 years?

**Participant A:** Yes

**Researcher**: Ok, as a lay counsellor the whole time?

**Participant A:** Lay counsellor

**Researcher**: Ok, so you know what you are doing

**Participant A:** Yeah I know what I am doing

**Researcher**: Ok and can you start to tell about your experience using Lynx as a…within the lay counseling

**Participant A:** Ok, when we started using the Lynx it was very difficult, but at the end of the day it was quiet easy to use the Lynx

**Researcher**: Ok, and what made it difficult at the beginning

**Participant A:** Because we didn’t know how to do it, when we are starting the thing we have to wait first so by the time we were given Lynx what you know is that all the time you start using the Lynx you know how to use it. So it was easy for us cos it was just to prep the client, yes

**Researcher**: Ok, so at the beginning you didn’t know how to do it so as you kept going you were able to use it?

**Participant A:** Yes

**Researcher**: Did you have training, or did you learn from other lay counsellors

**Participant A:** No they just came Madam Moronga* and the team they just got us as a team and the just told us to use the Lynx, but for me it was not very difficult because I used to use the laptop so I just follow the procedure. It was easy for me, because it was the 1^st^ time when they came but when I started it was very difficult buy at the end of the day it captures all the information

**Researcher**: Ok, that is good to hear and how has using it affected your work

**Participant A:** Come again?

**Researcher**: How has using the Lynx tablet affected recording patients as you test them or maybe has it affected the way that you would test them, just how has using the tablet affected the way that you work?

**Participant A:** No for me I said at first it was, but when I am doing my tests the clients see that I have to capture all the information through the client so that I enter in my phone, this tool is very effective.

**Researcher**: Ok, so then it must be different from just writing in the register

**Participant A:** It was easier it was just follow the…showing how to once you get it I just click the question comes just click like that

**Researcher**: Ok, so that’s good

**Participant A:** Yes

**Researcher**: And so does it take more or less time to do it on the tablet

**Participant A:** Just less than 4 to 5 minutes when you are entering, but when you are with client sometimes because you have to be patient asking the client questions, the client to answer the questions you have to enter into the Lynx wait with the client after the questions you have to wait 10 to 15 minutes

**Researcher**: Ok, that makes sense

**Participant A:** Yes

**Researcher**: And then do you still have to put clients in the paper register after you put them on Lynx

**Participant A:** Yes you have to enter in the Lynx and in the register

**Researcher**: Ok

**Participant A:** Yes to capture the information

**Researcher**: Ok, and which do you normally do first the paper or the Lynx, or does it depend on…

**Participant A:** First I have to do in the Lynx then enter in the register because you are with the client I have to get all the information because you are not supposed to enter in the Lynx alone you have to enter in the book because they have to see how you are working and to manage the data you have to count the things that you are using at the end of the month, so you are not supposed to enter in the phone alone you have to enter into the register

**Researcher**: Ok, so then is it different when you are capturing for a client in the facility versus in the community for Lynx?

**Participant A:** You mean when I go in the community to capture the in the Lynx for the clients?

**Researcher**: Yes

**Participant A:** No it’s not difficult because I am with the client I am able to ask the question she or he is able to answer

**Researcher**: Ok, so is it any different in the facility?

**Participant A:** There is no difference because even in the community you have to enter in the register

**Researcher**: Ok

**Participant A:** Yes, you have to write somewhere maybe in the book, when you come back in the facility you have to enter in the register

**Researcher**: Ok, and what about…there must be some differences during the day if it’s a very busy day at the facility or it’s a more quiet day how would you be maybe testing or capturing differently if it’s a busy day or if it’s a quiet day

**Participant A:** I have to manage to capture even if it is busy or not I have to do the work correctly because I am not supposed to just leave it like that I have to capture it in the Lynx so that the information they get perfect capture from me

**Researcher**: Ok, so what’s what sort of things would prevent you from being able to make the perfect capture from Lynx

**Participant A:** You mean, come again

**Researcher**: Like what sort of, is there anything maybe it happens during the day or with the tablet or with work that would make you not able to put in the Lynx

**Participant A:** Maybe if the Lynx is not working, you see sometimes this Lynx stops working when you are doing the directions this Lynx they stop, sometimes they don’t work they just stop

**Researcher**: Ok, do you know what would make them stop, like is it the battery or the network or…

**Participant A:** Sometimes they say it’s the network maybe there is no bundles and things like that

**Researcher**: Ok, but what ends while you have your responsibilities as a counselor would those ever prevent you from being able to put the capturing in?

**Participant A:** Like what?

**Researcher**: I mean I don’t know I am just asking

**Participant A:** Come again

**Researcher**: When you have your responsibilities as a counsellor because I understand that sometimes you are doing focus on one indicator and maybe another indicator at different works

**Participant A:** Yes

**Researcher**: Could that start to affect the time that you need to put into Lynx?

**Participant A:** I have to make sure that I do the right thing

**Researcher**: Ok, and you still manage

**Participant A:** Yes I manage for work I manage

**Researcher**: Ok, some of the other leaders I was discussing is that some times it’s too much happening

**Participant A:** Like if the Lynx is not ready I can’t but id the Lynx is ready I can manage

**Researcher**: OK, because I do also sometimes also see even for this hospital the numbers submitted to Lynx would numbers so maybe we say just like as an example last month in September lots of people were submitting for this facility but then maybe this month we come to October and I can see there is not so many people submitting but then maybe next month it goes back up again so do you know why some months its up and some months is low?

**Participant A:** Yes like people come at the facility

**Researcher**: But even regardless of how many people come, how many end up being captured on Lynx can change

**Participant A:** Yes sometimes what happens Sir sometimes people even like I said even in September we have some clients they come in test positive October they are changed so it’s like time changing, yes this month we have big number next month small number something like that, it’s like people are getting sick this month people have got too much there is sport there is too much I don’t know so people are just sick so sorhwy xomm

**Researcher**: Ok and then what about if there is a difference in the registers for the total tests completed versus the number on Lynx so if the registers are higher than the ones on Lynx sometimes on a different month so that we can see that not all of the tests ended up inside of the Lynx

**Participant A:** You mean in the registered are so many people but on the lynx they are only few?

**Researcher**: Yes so that on the register there are 20 but on the Lynx it’s only 8

**Participant A:** Yes its happening Like that

**Researcher**: So why would that happen

**Participant A:** Sometimes I can say maybe sometimes we have to 5 or 6 7 counsellor some they don’t have Lynx like that time the Lynx is not working for that person maybe 2 counsellors the Lynx are not working and 4 the Lynx is working so when you are capturing in the Lynx and in the register the so when you are capturing in the Lynx the number will be small but in the register the number will be big, there will be different

**Researcher**: And you are saying g it is mainly because the tablets don’t always work for every body

**Participant A:** Yes, sometimes they stop working they just stop, we can even try they can’t

**Researcher**: Ok can you think of anything else that might affect why they weren’t able to submit

**Participant A:** Sometime they said there is no internet, there is no bundles, .maybe the application has been changed I so no I don’t know something like that. So they need to make the Lynx to start working so that we could record and enter in the Lynx

**Researcher**: Ok, and what about when the Lynx is working, if it is working but we can still see that not as many there is only 12 in the Lynx now but there is still 20 in the register so we are still missing g some

**Participant A:** Yes maybe there is someone with us sometime there are so many categories that we are using, there are some couple testing, some …..some maybe they don’t capture it in the Lynx, they just at the end of the end maybe they just tested with out captured in the Lynx Maybe that can be the issue

**Researcher**: Which areas in the Lynx is normally used in?

**Participant A:** Everywhere

**Researcher**: Oh all of them

**Participant A:** Yes

**Researcher**: Ok, but then you think it maybe still some areas get Lynx more than the other ones?

**Participant A:** Yes

**Researcher**: Which ones do you think it’s used the most?

**Participant A:** The most indexing in the community and the facility

**Researcher**: Ok, index community and facility and Which ones you think it get used not as much maybe

**Participant A:** In the facility because sometimes they get to be busy they test 10 times maybe they enter in the Lynx maybe 4 or 5 something like that

**Researcher**: Ok, now that makes sense. We are almost done you are giving me the answers very fast and so that makes it easy. So how could you…could you maybe think of something to make it easier to submit on Lynx and in that for 2 one maybe in the tablet its self and one that maybe you hospital responsibilities are settle

**Participant A:** Maybe the other thing I can say for Lynx is that they send us the bundles I think they have to send us talk time then I supposed to make the bundles so that I know how to use because sometimes they send us bundles and you hope that bundles are there so by the time you are done using the Lynx it’s not there something like that

**Researcher**: Ok

**Participant A:** And make sure that the Lynx are well because sometimes they stop working

**Researcher**: Ok, so it’s the data bundles for the counsellors

**Participant A:** Yes and we need someone who can take care of the Lynx when they stop working so that we can capture

**Researcher**: Ok that makes sense. And do you have any other comments on Lynx or the weaknesses, what you like now what you don’t like about Lynx

**Participant A:** With the Lynx or….

**Researcher**: Just with the Lynx

**Participant A:** Sometimes I can say my comments from a health thinking I can say the Lynx they are good. But the they start using the Lynx then they stop so it could be that they become lazy, because not working then we stop using the Lynx and we have start using the Lynx late at the end of the month maybe in the month we use it once or twice the n it stops working

**Researcher**: Ok

**Participant A:** Yes so we don’t like that

**Researcher**: So when it suddenly stops?

**Participant A:** It was perfect

**Researcher**: Ok that makes sense

**Participant A:** That is comment I have

**Researcher**: Ok, it seems we are very good on time it’s been 15 minutes, I am trying to think if there is any other I want to ask you while you are here, time, resources we have cover it .

**Participant A:** Thank you very much
