# Supplementary material for: Health worker acceptability of an HIV testing mobile health application within a rural Zambian HIV treatment programme
Source: PLoS One. 2025 Jun 5;20(6):e0312646. doi: 10.1371/journal.pone.0312646 (PMC12140264; doi:10.1371/journal.pone.0312646)
Supplement: S10 File — (ZIP) [file pone.0312646.s010.zip › Transcript_11_deidentified.docx]

**Researcher**: Just to start can you go and you tell me how long have you been working as a counselor or how long have you been working at XX?

**Participant A:** More that 8 years

**Researcher:** Ok as a counselor?

**Participant A:** Yes

**Researcher**: And for you?

**Participant B:** Yes I started in 2008 for 14 years something like that

**Researcher**: Ok wow and for you?

**Participant C:** We started at the same period

**Researcher**: Also 2008?

**Participant C:** Yes we are senior citizens of this place

**Researcher:** So you must know everything about it?

**Participant C:** Yes

**Participant D:** Yes and me too in 2008 with the VATECT from the we moved to Equip and now we are almost 14 years

**Researcher**: OK, so you know this place well, I’m sure.

**Participant C:** Yes. For sure, she is the mother of this place. You can see.

**Researcher**: OK, maybe I will start this site this time. Can you tell me what your experiences are using while you were?

**Participant** **D:** While I was using it?

**Researcher**: Yes.

**Participant**: For the first time it was quite difficult for me but when I got used to it, it was very nice and the important thing was sending information direct. So we are sending information direct but after some years it has stopped working so when you try and try, the information was not sent.

**Researcher**: OK. Was there ever? Times when it stopped working and. Then they were able to. Fix it.

**Participant D:** They were able to fix it the last time when they fixed it. When they returned it back, it never worked again, so they third time. It’s when they have taken it without bringing it back to up to now.

**Researcher**: Ok, that’s too bad.

**Participant** **D**: We don’t have anything, we always complain. Sending information was very easy. Even if we are in the field, we can send information there. We have not reached the facility, we have not entered the register, but the information is already gone. So it was very important for me.

**Researcher**: OK, that’s good to hear. Now how about For you.

**Participant** **D**: Me the system was very good at the beginning it was so difficult since we were not familiar with the system. But later on when we caught up. It was working very nicely, although they were. The problem was sometimes you find that there was no Internet to connect to user Maria, that was the only challenge we used to face. Otherwise, the system was good.

**Researcher**: OK. How about for you?

**Participant C:** I can my colleagues said it earlier the system was very good. It was very easy even to send the information to the office was very useful. They have said It Here. that’s all. I’m just adding

**Researcher**: Do you agree with your colleagues or do you have something different or the same.

**Participant** **A**: Yes, it’s the same, but at the beginning it was working very well. But after that we used to record on the phone we had a breakdown of information until now we have nothing to record on the phone, the system was working very good..

**Researcher**: Ok, and can I ask, when the system was working still, how did it affect the way that you were either testing or cancelling? Did it make any changes? for the way that you were doing your test and counselling

**Participant D:** In fact, there was no changes.

**Participant C:** There was no change

**Participant B:** No changes.

**Participant D:** For the first Lynx that they gave us for the first time, it was a stretch forward for the second clinics, the system changed a little bit, yes.

**Participant C:** There were some areas where it wasn’t working. It was not matching with the first clinics.

**Participant B**: Like the landmarks, phone numbers, the client phone number. In the last clinics there was that indications, but the second clinic with our friends we were given there was that information, so as me, I got the first clinics. The second ones, no, I didn’t. I didn’t even touch it.

**Participant B:** Same as me

**Researcher**: The one with the phone numbers you didn’t have?

**Participant D**: No

**Participant C:** No these two didn't have there were three of us in fact

**Participant B**: The phones you bought us when you came the same phones we have.

**Participant D:** Same phones they took from us

**Participants C:** Me too we have the white ones me and this.

**Participant B:** Even this one. It’s only the new one the latest one

**Researcher**: Oh the tablet changed?

**Participant D:** Yes.

**Researcher**: OK.

( Inaudible)

**Researcher**: But the program should be the same

**Participant D:** No it was the same only some Indicators

**Researcher**: And when you were using the program, did that change the way you do cancelling or do testing if you didn’t have the program?

**Participant D:** No.

**Participant C:** No.

**Researcher**: So would you do the same sort of testing if you have the tablet, than if you didn’t have the tablet?

**Participant B:** We do. It’s just the same.

**Participant D:** We just get information from the client council them, test them. It’s just the same.

**Participant**: The only difference with the Lynx you just report direct.

**Participant B**:The same information we are reporting to the office.

**Participant D:** But now it’s just the register until we send the weekly report its when they see what we are doing.

**Researcher**: OK, would you use Lynx more when it was working? Would you? Use it more in the facility or community or both.

**Participant D:** Both the community and the facility.

**Participant C:** The community and the facility.

**Researcher**: Was it nicer to use it in the community or facility.

**Participant C:** Anywhere, either in the community or here in the facility.

**Participant D:** Both. When you are in the community, you are sending information before putting it in in the register. You come here, you enter in the register, but already you have sent it.

**Participant B:** So it’s just the same.

**Participant A:** It’s the same time direct information.

**Researcher:** Ok and would you use it less or more if it was busy at the facility, would you not do all the Lynx information.

**Participant B:** Even when you are home it’s easy.

**Participant D:** So when we are busy at the facility. You can send them at night when you are done.

**Participant C:** You just keep information and you do it in your free time.

**Researcher**: OK. Let me check on the other ones. So I know that there was difficult different tablets and they didn’t work always. Sometimes they would stop and would start to work later. But when it was working, is there anything that you would want to improve on the way that it was working? Or even like a change to your work schedule to make it easier or something like that.

**Participant D**: Yes, we would want it to be changed like our friends who are from ( inaudible) unit they are connected to WhatsApp. They are sending their own information through WhatsApp, having meetings they do have. But as we don’t have.

**Participant A**: Like change everything they can know

**Participants C**: So the communication system is changing and we need the system to improve.

**Researcher:** Which communication system?

**Participant C:** As you are saying, the Internet, everything is there, you just work your need, you need. But if you just rely on the whole system, we need it to be improved.

**Researcher**: OK improved how? I’m confused as to which system exactly like you.

**Participant C**: Ok,You said at first. You. Would like to change the system or not? The Lynx system.

**Researcher**: The Lynx system yes.

**Participant** **C**: …the Lynx system is if there is an improvement. Somewhere in the system you could like to be somewhere with our friends where they are. That work they have that work tablet.

**Researcher**: OK, so if there is a tablet. a new tablet somewhere here everyone else needs to get the same new tablet

**Participant D**: Yes.

**Participant C**: Yes.

**Participant A**: That would make it easy to use.

**Participant D:** Another (inaudible)

**Participant B**: Even the meetings. We are having meetings every Friday, right? Yeah. Only using the supervisor’s phone to link to the head office there in Gasa to get in touch with them, but we got our phones, we can even if we only counselors we can even link to that and get information and have a meeting with them

**Participant B:** And we can even talk with you on video what?

**Participant D**: Video calls, yes.

**Participant B**: video calls.

**Participant C:** And we can report daily at the same time. It can be easy.

**Researcher**: OK, makes sense. And again, when you were using it, would it work better or worse when the day was busy? If it was a busy day.

**Participant B**: Come again.

**Participant C**: We can find the time when we are free so that we can report.

**Participant B**: The system was very easy

**Participant D:** No, we are not always busy when you report at the week there is a time , like lunch time don’t go anyway. If you were in the community, we used the client. You see the client and you find the time. You can start reporting there. You finish reporting.

**Participant B:** Sir can you repeat that question again? We are lost.

**Researcher**: I mean, I think they answered, but for me I was just more wondering if it was extra hard or?

**Participant B:** As I said earlier it was easy, was very easy, very easy.

**Researcher**: OK, that’s good to hear, ok so I can also see sometimes because I see XX always did submit when I was working but some months I can see that everyone submitted lots of test and then maybe the next month it would go down but then the following month everyone is submitting again months not too many, do you know why some months…?

**Participant C**: We are not submitting?

**Researcher**: As much as other months yes

**Participant C:** The reason for that we had some challenges sometimes when the tablets…

**Participant D:** Stop working.

**Participant C:** Stop working. When they take it to the office, but for those people to bring it back, it takes ages. That’s the only thing.

**Participant D**: It would be two months. When they bring it back, they start working with it, it stops. Then you just pack it.

**Researcher**: OK, but I think I saw it up and down when it should have still been working here. Before it was in the office.

**Participant B**: No sir we are working we are reporting

**Researcher**: No I am not here to get you in trouble I want to…

**Participant C**: No we are here to speak what ever that is the truth we used to report…

**Participant D**: Weekly report, monthly report but mainly we are doing daily reports daily reporting when you finish work you report

**Participant B**: At the end of the week we report

**Participant D:** Maybe Saturday or Sunday when we are not working then we don’t report anything

**Researcher**: Sure sure?

**Participant D**: Yes

**Researcher**: So is there anything…but for when it’s working I know that you do need to have better IT support so that they can fix it faster and also make sure that everyone has the same Lynx tablet if he has a nicer new one everyone has the same or some facilities that also get

**Participant** **C:** That is true Sir

**Researcher:** So what else could be done to make it also better to use

**Participant C:** The only thing….the only challenge we used to have is when the system the Lynx had a problem when the got there it took centuries for those people to bring back the Lynx back

**Participant D:** But at this moment if we are given we are sure we will be reporting

**Participant C:** And some of us it has even taken so long we can’t it needs for us to go through it to be oriented on the same thing

**Participant A:** But we can use we can try

**Participant B**: Now before that we need just a short orientation

**Participant C:**…short orientation first because we have taken so long

**Participant D**: We knew it to do it but we have forgotten some how some where that’s why we need it we have forgotten some how somewhere but we can be making these things or we can when you say I have

**Participant B:** Got this I am doing this it just a matter of 5 minutes we need a refresher course to remind ourselves to start again

**Researcher**: You can tell your boss that they must bring me back, I can come back again

**Participant D:** Yes we support you come back

**Researcher**: Ok those were all of the questions I have is it ever one more is it ever I know sometimes you have different work responsibilities like sometimes you focus on PreP and sometime you focus on dispensation or maybe even TB so if the program is asking you to focus on something different how would that affect the way you….would that affect the way you would do your counseling like would that make you do it more on the register or..?

**Participant D:** Yes

**Participant C:** That one you are right it affects us

**Participant D:** It affects us because the TB counselors they know how to do it about TB but us we focus on HIV, so HIV binding index, theatrics, PMTCT and HIV testing

**Participant C**: (inaudible) that on that you have said that the madam have said another thing it’s another department for those people to give us information from them there is some slight difference

**Researcher**: Sure

**Participant C:** Yes

**Participant B**: The people who are on PreP sometimes there is a problem, like shortage of PreP so when clients come we say we haven’t got anyone so we have told them at the office we have a shortage no PreP long time 5 months, 6 months we are waiting (inaudible)

**Participant D:** But the thing is when they changed the system they tried to orientate us but we cannot disagree with what they want us to do so we are here to obey what they want us to do when they changed the system they know (inaudible) is working on this Elizabeth you are working on this, we shall be orientated

**Researcher**: Ok it all makes sense, ok that’s good

**Participant B:** Because it’s out responsibility

**Participant C**: And during the your time…

**Participant B:** Yes you know what I mean sir

**Researcher**: I know what you mean

**Participant C**: And during your time it wasn’t taking time as it used to do these days when you get it, it was easier for you just a week

**Participant D:** Or a day

**Participant C**: Or a day you bring them back, but since you left it has been a challenge

**Researcher**: I mean things change in the program I am not based in Katsanga anymore I am in South Africa because they want local people here to run the program

**Participant C**: Maybe they are not all that experienced with the system

**Researcher**: it’s ok, I mean we are learning

**Participant C:** Though the challenge we are facing when it comes to our hands we are local failures and its another story

**Participant D:** So sir that’s all we have

**Researcher**: Ok anymore from you two

**Participant B:** That is another business Sir we don’t have bags for…

**Researcher**: Ok maybe you can tell that to Mr. Roger’s because I am not in charge as I said I don’t work here anymore, I can tell him but…

**Participant D:** Its not necessary

**Researcher**: He won’t (inaudible)

**Participant D**: As we are trying to say it’s about the Lynx

**Participant A**: We want the Lynx

**Researcher**: Ok it’s good to hear I can see that it is easy to use we just have to make sure that the tablets are working for everyone and if something happens to the tablet…

**Participant B**: ( inaudible) Why us in XX we don’t have the Lynx

**Researcher**: That is true even in (inaudible)

**Participant B:** But we do the same job

**Participant D**: But we do the same reports so why us here in XX we don’t have the Lynx

**Researcher**: I don’t know

**Participant D**: I am just asking, I am just asking why us here they don’t give us Lynx why? So for information we need it please.
