# Supplementary material for: Health worker acceptability of an HIV testing mobile health application within a rural Zambian HIV treatment programme
Source: PLoS One. 2025 Jun 5;20(6):e0312646. doi: 10.1371/journal.pone.0312646 (PMC12140264; doi:10.1371/journal.pone.0312646)
Supplement: S5 File — (PDF) [file pone.0312646.s005.pdf]

Plot No. 1, Cnr Joseph Mwilwa & Great East Road
Rhodes Park, Lusaka - Zambia
Tel: +260 955 155 633
+260 955 155 634
Cell: +260 977 493220
Email: eresconvergeltd@gmail.com

I.R.B. No. 00005948
F.W.A. No. 00011697

29th October, 2021.

Ref. No. 2021-Oct- 003

The Principal Investigator
Dr.Cheswa Vwalika
Plot 22877 leopards Hill Road, Ibex Hill
LUSAKA, ZAMBIA

Dear Dr. Vwalika

REF: END TERM EVALUATION OF THE EXTENDING QUALITY IMPROVEMENT PROGRAM (EQUIP)

Reference is made to your protocol resubmission. The IRB resolved to approve this study and your participation as Principal Investigator for a period of one year.

Review Type	**Fast Track**	Approval No. **2021-October-003**
Approval and Expiry Date	Approval Date: 29th October, 2021	Expiry Date:
Protocol Version and Date	Version - Nil.	28th October, 2022
Information Sheet, Consent Forms and Dates	• English.	28th October,2022
Consent form ID and Date	Version - Nil	28th October,2022
Recruitment Materials	Nil	28th October,2022
Other Study Documents	Questionnaire.	28th Octobe,2022
Number of participants approved for study	-	28th October,2022

Specific conditions will apply to this approval. As Principal Investigator it is your responsibility to ensure that the contents of this letter are adhered to. If these are not adhered to, the approval may be suspended. Should the study be suspended, study sponsors and other regulatory authorities will be informed.

Conditions of Approval

- No participant may be involved in any study procedure prior to the study approval or after the expiration date.
- All unanticipated or Serious Adverse Events (SAEs) must be reported to the IRB within 5 days.
- All protocol modifications must be IRB approved prior to implementation unless they are intended to reduce risk (but must still be reported for approval). Modifications will include any change of investigator/s or site address.
- All protocol deviations must be reported to the IRB within 5 working days.
- All recruitment materials must be approved by the IRB prior to being used.
- Principal investigators are responsible for initiating Continuing Review proceedings. Documents must be received by the IRB at least 30 days before the expiry date. This is for the purpose of facilitating the review process. Any documents received less than 30 days before expiry will be labelled "late submissions" and will incur a penalty.
- Every 6 (six) months a progress report form supplied by ERES IRB must be filled in and submitted to us.
- A reprint of this letter shall be done at a fee.

Should you have any questions regarding anything indicated in this letter, please do not hesitate to get in touch with us at the above indicated address.

On behalf of ERES Converge IRB, we would like to wish you all the success as you carry out your study.

Yours faithfully,
ERES CONVERGE IRB

Dr. Jason Mwanza
Dip. Clin. Med. Sc., BA., M.Sc., PhD
CHAIRPERSON
