# Supplementary material for: Health worker acceptability of an HIV testing mobile health application within a rural Zambian HIV treatment programme
Source: PLoS One. 2025 Jun 5;20(6):e0312646. doi: 10.1371/journal.pone.0312646 (PMC12140264; doi:10.1371/journal.pone.0312646)
Supplement: S7 File — (PDF) [file pone.0312646.s007.pdf]

Plot No. 272, Cnr Olive Tree Meanwood Road,
Meanwood Ibex
Lusaka - Zambia
Tel: +260 955 155 633
+260 955 155 634
Cell: +260 977 493 220
Email: eresconverge@yahoo.co.uk

I.R.B. No. 00005948
F.W.A. No. 00011697

19th September, 2022.

Ref. No. 2021-Oct- 003

The Principal Investigator
Dr.Cheswa Vwalika
Plot 22877 leopards Hill Road, Ibex Hill
LUSAKA

Dear Dr. Vwalika

RE: AMENDMENT, RENEWAL, END OF YEAR AND 6 MONTHS PROGRESS REPORT FORM: END TERM EVALUATION OF THE EXTENDING QUALITY IMPROVEMENT PROGRAM (EQUIP)

We would like to acknowledge receipt of your end of year progress, 6 months and Amendment report dated 15th September, 2022.

The study is extended for another year. The new expiry date is 14th September, 2023.

Yours faithfully,
ERES CONVERGE IRB

Dr. Jason Mwanza
Dip. Clin. Med. Sc., BA., M.Sc., PhD
CHAIRPERSON

19th September, 2022.

Ref. No. 2021-Oct- 003

The Principal Investigator
Dr.Cheswa Vwalika
Plot 22877 leopards Hill Road, Ibex Hill
LUSAKA

Dear Dr. Vwalika

RE: AMENDMENT, RENEWAL, END OF YEAR AND 6 MONTHS PROGRESS REPORT FORM: END TERM EVALUATION OF THE EXTENDING QUALITY IMPROVEMENT PROGRAM (EQUIP)

We would like to acknowledge receipt of your end of year progress, 6 months and Amendment report dated 15th September, 2022.

The study is extended for another year. The new expiry date is 14th September, 2023.

Yours faithfully,
ERES CONVERGE IRB

Dr. Jason Mwanza
Dip. Clin. Med. Sc., BA., M.Sc., PhD
CHAIRPERSON
